# Glass import and production in Hispania during the early medieval period: The glass from Ciudad de Vascos (Toledo)

**DOI:** 10.1371/journal.pone.0182129

**Published:** 2017-07-26

**Authors:** Jorge de Juan Ares, Nadine Schibille

**Affiliations:** IRAMAT-CEB, UMR 5060, CNRS, Orléans, France; New York State Museum, UNITED STATES

## Abstract

One hundred and forty-one glass fragments from medieval Ciudad de Vascos (Toledo, Spain) were analysed by laser ablation inductively coupled plasma mass spectrometry (LA-ICP-MS). The glasses fall into three types according to the fluxing agents used: mineral natron, soda-rich plant ash, and a combination of soda ash and lead. The natron glasses can be assigned to various established primary production groups of eastern Mediterranean provenance. Different types of plant ash glasses indicate differences in the silica source as well as the plant ash component, reflecting changing supply mechanisms. While the earlier plant ash groups can be related to Islamic glasses from the Near East, both in terms of typology and composition, the chemical signature of the later samples appear to be specific to glass from the Iberian Peninsula. This has important implications for our understanding of the emerging glass industry in Spain and the distribution patterns of glass groups and raw materials. The plant ash that was used for the Vascos glasses is rich in soda with low levels of potash, similar to ash produced in the eastern Mediterranean. It could therefore be possible that Levantine plant ash was imported and used in Islamic period glass workshops in Spain. Unlike central and northern Europe where an independent glass industry based on potassium-rich wood ash developed during the Carolingian period, the prevalence of soda ash and soda ash lead glass on the Iberian Peninsula indicates its commercial and technological interconnection with the Islamic east. Our study thus traces several stages leading to the development of a specifically Spanish primary glassmaking industry.

## Introduction

Recent research on the chemistry of ancient and medieval glass has established compositional groups that are linked to the materials' origin and date of production. For most of the first millennium CE, the overwhelming majority of glass from the Mediterranean and central Europe was manufactured on the Levantine coast and in Egypt, using Egyptian natron as the main fluxing agent [1, 2], although the geochemical characteristics of some early Roman glass raise the possibility of a western Mediterranean provenance [3]. In the eighth century, mineral natron was gradually replaced by vegetable fluxes: soda-rich plant ash in the Islamic world, and potassium-rich wood ash in central and northern Europe. Around the same time, high lead glasses also appear, both in Europe as well as in the Islamic east [4–6]. Little is known about glass from the Iberian Peninsula, mainly due to the small number of analyses carried out to date. Natron-type glasses of known compositions have been identified, but their regional and chronological distributions remain unclear. Similar to the eastern Mediterranean, vegetable soda glass had replaced natron glasses by the tenth century, but at what point exactly this transformation took place and where these soda plant ash glasses came from is uncertain [7]. Systematic large-scale studies of well-dated assemblages are needed to better understand the distribution patterns of glass in Iberia.

The present article reports the chemical data of 141 glass samples from Ciudad de Vascos (Navalmoralejo, Toledo) next to the river Tajo in Central Spain [8]. In the tenth and eleventh century CE, Ciudad de Vascos was a small fortified urban settlement with mosques, baths and cemeteries on the border of Islamic al-Andalus and the Christian kingdoms to the north. The archaeological excavations additionally yielded Roman and early medieval remains, such as ceramics, construction materials and epigraphic stones [9], as well as a substantial number of late Roman coins dated to the reigns of the Roman emperors from Constantine to Honorius (303–423 CE). Hardly any material can be unambiguously attributed to the Visigothic or the earlier Islamic period. After the Christian conquest of Toledo in 1085 CE, a small Christian settlement, limited to the kashbah, existed until the first decades of the twelfth century, after which the site was abandoned [10, 11]. The stratigraphic sequence is more or less preserved from the middle of the ninth and tenth centuries and most of the vestiges of occupation date, in fact, to the eleventh century. The various archaeological materials (metals, ceramics, lithic, glass, etc.) have been widely published, making the finds from Ciudad de Vascos the best-known repertoire of tenth- and eleventh-century material from central Iberia.

The archaeological finds from Ciudad de Vascos allow us to trace some of the chronological developments and commercial networks of medieval Spain, about which our knowledge is still limited. The numismatic finds from the early Islamic period of the Umayyad Emirate of Cordoba, for instance, are very scarce. No more than a couple of coins recovered from Vascos date to the eighth and ninth centuries. Caliphal tenth-century coins from the mints of Cordoba are likewise very limited. The vast majority of coinage date to the second half of the eleventh century, minted mostly at Toledo in the name of al-Maʻmun (1043–1075 CE), al-Qadir biʻllah (1074–1090) and, since 478-479H (1085–1086), in the name of Alfonso VI. The coins from other locations are less represented, but some have been identified from Cuenca (1081) and Valencia (1061–1064), both by al-Maʻmun, from Badajoz by al-Mutawakkil (1072–1094), Seville by al-Muʻtamid (1068–1091), and from León by Alfonso VI (1072–1109), probably a Fatimid fragment, as well as some coins of uncertain provenance from Alpuente and Ceuta dating to the late eleventh century [11–13].

The scientific analysis of the glass from Ciudad de Vascos can provide further information on the site’s cultural and economic connectivity during the tenth and eleventh centuries CE. Here we present new analytical results of a substantial set of samples, thus producing quantifiable data about the transmission of goods, both on a local as well as long distance scale between medieval Spain and the wider Mediterranean world. The analytical results reveal distinctive patterns of production and consumption, documenting the technological and historical developments in central Spain. There is vitreous evidence for the late Roman occupation of the site in the form of common late antique glass groups originating in the eastern Mediterranean. A clear interruption during the Visigothic and early Islamic period is evident from the absence of early Islamic glass groups, while a certain degree of prosperity during the tenth and eleventh centuries is reflected in the presence of several distinct plant-ash glasses. As will be discussed, the earlier plant ash glasses show similarities with examples from the eastern Mediterranean and may be imports, whereas the later ones were possibly made somewhere on the Iberian Peninsula.

## Materials and methods

### Archaeological context and samples

**Fig 1:**
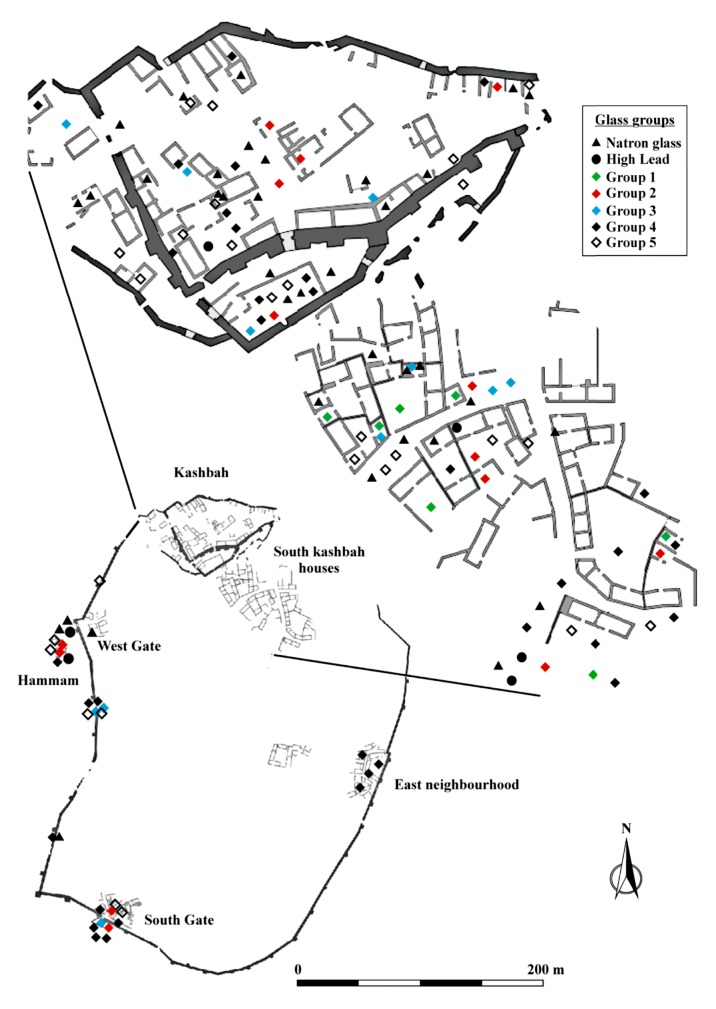
Spatial distribution of chemical glass groups in Ciudad de Vascos.

Over the entire forty-year period that excavations have been carried out at Ciudad de Vascos, only about 165 different glass objects have been recovered, 141 of which were sampled and analysed by laser ablation inductively coupled plasma mass spectrometry (LA-ICP-MS) for the present study. The glass fragments are stored at the Santa Cruz Museum in Toledo (Spain) and were sampled and analysed with the official authorization from the Consejería de Educación, Cultura y Deportes de la Junta de Comunidades de Castilla-La Mancha, Spain. The material comes from different stratigraphic contexts and from different find-spots throughout the archaeological site (Fig 1). Unfortunately, the intense occupation of Ciudad de Vascos during the Islamic period altered the previous stratigraphy and prevents well-defined chronological attributions of the pre-Islamic and earlier Islamic material except for a few fragments. The stratigraphy of the tenth and eleventh century, in contrast, allows for the distinction of chronological trends (S1 Table). Typological considerations can furthermore help in establishing a relative chronology of the finds. For example, practically all glasses of the natron family can be attributed to pre-Islamic types such as goblets and glasses of greater thickness. Some of the vessel types circulating during the Islamic period include beakers, bottles and unguentaria as well as several pieces of decorative sheet glass (S1 Table). Most of the glass is free blow with a limited presence of mould-blown samples. Decorations are restricted to very few fragments like unguentaria with applied trail decorations, a few examples of scratch-engraved ornaments and mould decorations that have parallels in glass objects from the Near East. The assemblage exhibits a relatively wide chromatic variety with a preponderance of colourless and weak colours (greenish, bluish, yellowish) and a few strong colours such as blue, green and purple [14].

### Analytical method

Small samples of 3 mm size were mounted in epoxy resin and polished to remove any surface contaminations and corrosion layers. The mounted and polished sections were then analysed by LA-ICP-MS at IRAMAT-CEB, following the same protocol as described in [15–17]. The mass spectrometer used is a Thermofisher Element XR and the laser ablation system is a Resonetic UV laser microprobe (193 nm Excimer laser). The operating conditions were set at 5 mJ with a frequency of 10 Hz and a spot size diameter of 100 μm that was reduced where necessary to avoid saturation. Pre-ablation time of 20 seconds was followed by 40 seconds analytical time, measuring fifty-eight elements, including all major constituents of archaeological glass, and various minor and trace elements (S1 Table). Quantitative results were calculated based on the principle of an internal standard and a set of international glass standards (Nist SRM610, Corning B, C and D) as well as an archaeological glass of known composition (ALP1) for the determination of chlorine [16]. Glass standards NIST SRM612 and Corning A were analysed at regular intervals to establish the accuracy and precision of the analyses (S2 Table). The analytical precision is better than 5% relative for most elements with very few exceptions among some of the trace and rare earth elements. The accuracy of most of the major and minor elements is within 5% relative, and better than 10% for most of the trace elements (S2 Table). The calcium values deviate more substantially in the Corning A standard (> 10%), but show a much better consistency with the published values for NIST SRM612 (< 4%). The detection limits vary between 0.1 and 0.01% for major elements and between 20 and 500 ppb for trace elements, depending on the analytical parameters [16].

## Results

**Fig 2:**
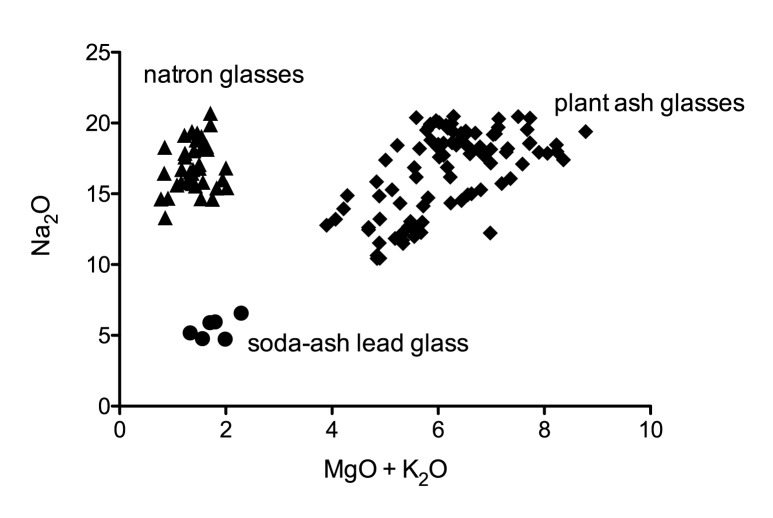
Soda concentrations versus the sum of potash and magnesia of all glasses from Ciudad de Vascos. The three main glass types are easily distinguished based on the sum of the alkali and alkaline earth components MgO and K_2_O.

All 141 glasses analysed from Ciudad de Vascos are soda lime silica glasses that fall into three main compositional groups, according to the use of different fluxing agents (S1 Table, Fig 2). By far the largest group (93) consists of soda-rich plant ash glasses, typical of Islamic glass from the eighth/ninth century onwards [18–20]. This type of glass seems to be present on the Iberian Peninsula since at least the ninth century [7, 21]. The second largest group represented at Vascos (37) are natron-type glasses of the Roman tradition [22]. Finally, there is a small set of six fragments with high lead contents (40–48%), similar to glasses previously described in medieval Islamic contexts [4–7, 23]. Five samples are classified as outliers, because they show unusual compositional features and cannot be attributed to any of the groups with certainty. Two samples (VS021 & VS027) have exceptionally high tungsten levels that cannot be easily explained. VS027 has together with samples VS012 and VS092 the highest potash concentrations. Sample VS091 seems to be a relatively typical natron glass except for its elevated magnesium concentration (S1 Table). The five outliers are not considered further.

### Natron glasses

The samples of this family are characterised by their low potassium and magnesium oxide levels (<1.5%) and high soda concentrations [24]. The comparison of the samples from Ciudad de Vascos with known natron glass groups reveals a great diversity with the presence of at least five different types: HIMT / Foy-1, Foy-2, Levantine, Roman Sb and Egypt II.

#### HIMT / Foy-1

**Fig 3:**
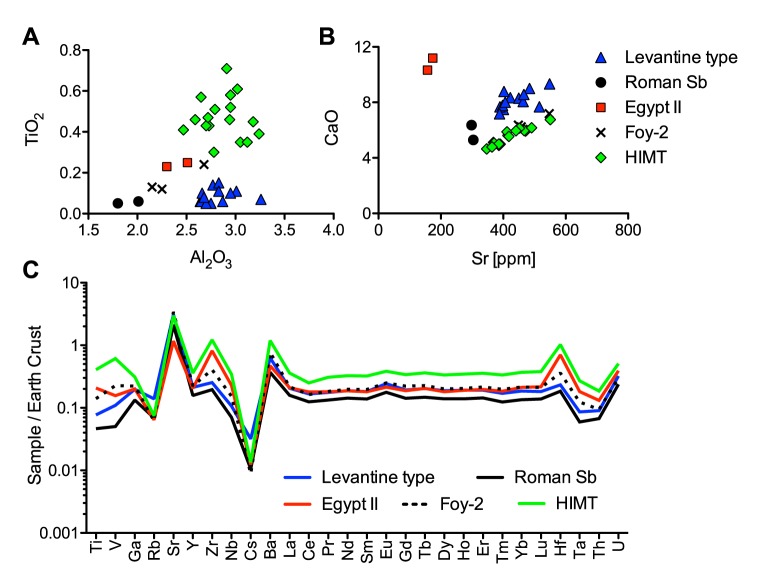
Compositional differences related to the silica sources of the natron glasses from Vascos. (a) Titanium versus aluminium contents highlight differences in heavy element contents of the different glass groups; (b) different strontium to lime ratios imply different sources of calcium for Levantine I, HIMT and Egypt II; (c) means of selected trace element and REE data of the different natron groups normalised to the continental crust (MUQ [30]).

Almost half of the natron glass is made up of so-called HIMT [25] or ‘série 1’ [1] (S1 Table). Either greenish or amber-yellow, it is characterised by high iron, manganese and titanium contents as well as elevated trace and rare earth elements (REE), while lime levels are relatively low (CaO < 7%) (Fig 3). The production location of these glasses is unknown, but the relative proportions of major and trace elements as well as the isotopic signatures for neodymium, lead, oxygen and strontium suggest an Egyptian provenance [1, 26, 27]. HIMT / Foy-1 was widespread during the fourth and fifth centuries (Albania, Bulgaria, Cyprus, Egypt, France, Italy, Tunisia, and the United Kingdom), but it seems to have been conspicuously absent from the Syrio-Palestinian area [1, 28, 29] (and references therein). On the Iberian Peninsula, it has been identified in Portugal, Extremadura, Madrid, Catalonia, Cuenca, Valencia, Murcia, Leon and in Seville [7].

#### Levantine-type glass

Thirteen samples have compositional characteristics of glass produced from a Levantine silica source [18, 19, 31–34], with relatively high lime and alumina and low heavy elements such as titanium, iron and zirconium (Fig 3). Compared to the HIMT / Foy-1 group, the Levantine glasses are more depleted in REE (Fig 3C). The Levantine-type glass from Vascos possibly includes both the so-called Levantine I group, related to the glass produced in the sixth- to seventh-century workshops of Apollonia [18, 19, 32], and earlier glasses from Jalame [31] as well as Roman manganese decolourised glasses [1, 5, 33, 34]. Given a certain degree of recycling manifest in the contamination levels of these glasses (S1 Table) and the poor chronological resolution of the samples in this group, it is not possible to separate them strictly into Roman and Levantine I. In Hispania, Levantine glass has been found in Tarragona [35] as well as Guadalajara (Recópolis) (in preparation).

#### Miscellaneous

The remaining seven fragments encompass Roman antimony, Foy-2 and Egypt II types. Two samples (VS057, VS097) appear to be related to the Roman antimony group even though one of the two (VS097) has no antimony (S1 Table) [5, 33, 34, 36, 37]. They both have low concentrations of lime, alumina and heavy elements as well as the lowest REE levels (Fig 3). This glass type has been linked with an Egyptian origin because of its high soda levels and TiO_2_ to Al_2_O_3_ ratios as well as its prevalence in Egypt [1, 34, 37, 38]. It has been proposed that Roman Sb glass was referred to as the glass from Alexandria in the Price Edict of Diocletian [34, 39], but no primary production centre has up to now been archaeologically ascertained. It is found throughout the Roman Empire and generally dates to the first to fourth century CE [33, 34]. In Iberia, it has been identified among glass assemblages from central and northern Portugal, in Leon and in Asturias [7].

Three samples correspond to the so-called Foy-2 production group (série 2.1), also known as HLIMT, weak HIMT or HIMT2 [1, 17] (and references therein). It has somewhat higher aluminium, titanium, iron, sodium and magnesium oxide concentrations than Levantine glass, but lower values than HIMT across all these elements (Fig 3). This compositional group was supplied to secondary workshops throughout the Mediterranean and raw glass chunks were found in France (Montpellier, Marseille, Vendres and Bordeaux) [1]; Bulgaria (Serdica) [40] and Tunisia (Carthage) [34]. Iberian workshops used raw glass of the Foy-2 composition in Lugo, Vigo [41] and Recópolis (in preparation). Foy-2 glass is commonly dated to the sixth to seventh century CE [17, 34].

Interestingly, two samples (VS018, VS031) correspond to Egypt II [19, 42]. This is the first time that Egypt II has been identified in Spain. One of the samples is dated on grounds of the stratigraphic sequence before the second half of the tenth century CE. It is a bottle fragment with a funnel-mouthed rim recovered from a small neighbourhood mosque [43]. Bottles of this type are well known in the Near East from the Abbasid-Fatimid periods, for example, from Ramla, Caesarea, Tiberias and Beth Shean, and they can probably be attributed to an Early Islamic date [44–46]. No primary workshop of Egypt II glass is known, but the high proportions of iron, titanium, zirconium and niobium suggest an Egyptian origin (Fig 3). Commensurate with the characteristics of Egypt II glasses, the two samples from Vascos have high amounts of calcium, low aluminium and low strontium characteristic of calcareous rocks instead of sand enriched with shells, thus indicating the use of a continental silica source [47]. The precise chronology of this glass has been established through the analysis of Abbasid glass weights from Fustat to 750 to 868 CE [42]. Glasses of this composition have since been found in Raya in South Sinai [48], in the seventh- to eighth-century secondary workshops of Khirbet al-Ḥadra (Israel) [49], in the ninth-century contexts of El Ashmunein (Egypt) [50] and San Vincenzo al Volturno (Italy) [51].

### Soda plant ash glass

With 93 soda plant ash glasses the study of the assemblage from Ciudad de Vascos represents the largest set of plant ash glasses ever published from the Iberian Peninsula. Most fragments are dated to the tenth and eleventh century CE, with some dates confined to within half a century (S1 Table). The glass is mostly colourless or weakly coloured, with some exceptional dark blue, purple or green samples. Their high levels of soda and low potash (S1 Table, Fig 2) are congruent with ash obtained from soda-rich halophytic plants from saline semi-arid or coastal environments [52, 53]. The lime concentrations are highly variable (S2 Table), due to a combination of factors. The calcium content of plant ash varies substantially [54, 55] and can be modified through purification processes [56]. Additionally, calcium can also partially derive from the silica source. As is quite common in Islamic glasses, the plant ash glasses from Vascos have elevated manganese contents (MnO > 0.5%), indicating its deliberate addition as decolouring agent [57].

#### The silica groups

**Fig 4:**
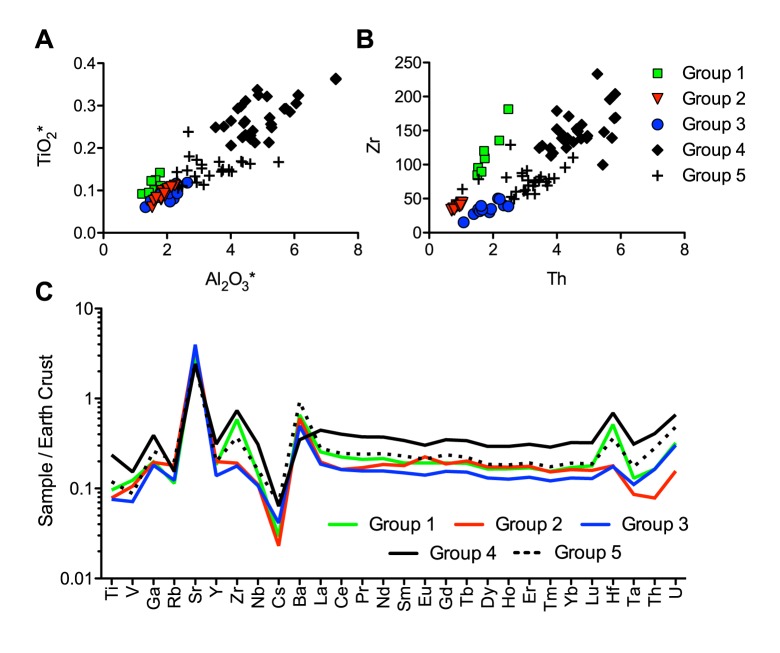
Elements associated predominantly with the silica source of the plant ash glasses from Vascos. (a) Titanium and aluminium concentrations identify different silica sources; (b) different correlations of zirconium and thorium separate groups 1, 2 and 3 more clearly; (c) means of trace and REE data of the five different plant ash silica groups normalised to the continental crust (MUQ [30]) confirm variations in the silica source.

The compositional variability of plant ash makes the interpretation of this type of glass more complex than that of natron glasses. Nonetheless, when considering elements that are (almost) exclusively introduced as part of the silica source such as titanium, aluminium, zirconium and thorium, five different groups can be differentiated among the Vascos plant ash samples (Fig 4A and 4B). These groupings are confirmed by distinctive trace element and REE patterns (Fig 4C). Intriguingly, the archaeological analysis shows furthermore the existence of chronological, typological and functional differences between the compositional groups.

Group 1 is the smallest group with only seven fragments, mostly colourless and belonging to flat or sheet glass, some of which are cut into regular pieces, possibly indicating a decorative function [14]. All these fragments derive from domestic contexts south of the citadel, possibly the richest area in the city (Fig 1). The composition of these samples is characterised by relatively low levels of alumina, titanium and niobium combined with elevated heavy elements zirconium and hafnium (Fig 4). The different ratios of zirconium to niobium and titanium point to the use of a silica source different from the other plant ash groups. All glasses of group 1 have small amounts of contaminants such as lead and copper (< 0.5%), suggesting that these glasses have undergone some degree of recycling.

Groups 2 and 3 seem closely related, except for varying zirconium to thorium ratios as well as vanadium and yttrium levels (Fig 4). They both have aluminium and titanium values similar to group 1, while group 2 has the lowest thorium, tantalum and uranium. Plant ash group 3, in contrast, has the lowest overall trace elements and REEs (Fig 4C). The glasses of group 2 and 3 were found mostly in areas that generally contain the oldest deposits from the early Islamic occupation, namely in the citadel and surrounding houses and baths. The samples are predominantly colourless or have a slightly green and blue tinge, and some samples have been intentionally coloured through the addition of copper or lead (S1 Table). Group 2 entails small tubular flasks, bottles and almost all mould-blown and engraved vessels. Group 3 shows a wide typological diversity, it was used in jewellery, bottles, unguentaria, dishes and goblets, one of which is decorated with circular boss at the centre (VS093) (Fig 2.19 [14]) characteristic of some Near Eastern glasses (e.g. No. 123 [58]).

The samples of group 4 make up about a third (31 samples) of all the soda plant ash glasses with a very distinctive chemical make-up (Fig 4). The sand source utilised for these glasses was rich in accessory minerals such as feldspar, magnetite, monazite, sphene and zircon as judged by the elevated levels of aluminium, iron, niobium, titanium, yttrium and zirconium, as well as higher REE contents relative to the other plant ash groups. In fact, all samples of group 4 have alumina contents in excess of 3.5%, which is unusual for contemporary plant ash glasses from the eastern Mediterranean and European sites with the exception of Italy, Spain and Portugal [59–64]. To this group belong fragments of tubular unguentaria, some with applied decoration, as well as some small bottles, fragments of lamps and a single piece of decorative flat glass. Based on the archaeological context the samples of group 4 date to the eleventh century CE, although for some of the fragments an earlier, tenth-century date, cannot be ruled out.

**Fig 5:**
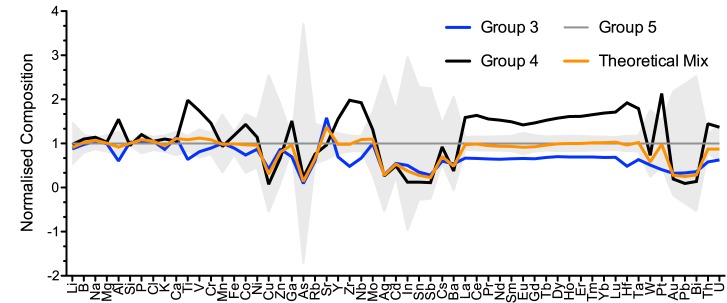
Computation of a mixed glass composition corresponding to group 5. Averages of the group 3 (n = 12) and 4 (n = 31) compositions were normalised to the mean of group 5 (n = 28). A theoretical composition (groups 3 and 4 at a mixing ratio of 2:1) was calculated and normalised to group 5. Grey areas represent the standard deviation of the group 5 composition.

An intermediate composition characterises the glasses of plant ash group 5, with aluminium, titanium, thorium, zirconium, trace and rare earth element contents all situated between groups 3 and 4 (Fig 4). It is a relatively heterogeneous group that might result from the recycling and mixing of other plant ash glasses. That the mixing of groups 3 and 4 could result in the group 5 composition, can be mathematically simulated (Fig 5). First, the theoretical mixing ratio was estimated by calculating the relative deviations of groups 3 and 4 from the group 5 composition for each element and the median was determined (median ratio of group 3 to group 4 is 2:1). Then, a theoretical mixed composition was calculated and compared to the analytical data of group 5 (normalised data). The hypothetical glass composition closely matches that of group 5 as regards the base glass components (orange trace in Fig 5). It differs in terms of the colouring agents such as copper, tin, antimony and lead and associated minerals, probably because of recycling. Four samples have lead contents of up to 8% and all but one are deep blue, turquoise or green (S1 Table). This atypical composition may be indicative of colouring techniques similar to those described in Islamic sources [4] that specify the addition of moderate amounts of lead (from 4% to 8%) and copper (> 1.5%). The remaining samples of group 5 are mostly colourless or have a blue, green or purple tinge. This type of glass was used for small bottles and unguentaria, a few bowls and jewellery. Numerous samples of group 5 were retrieved from archaeologically well-stratified contexts dating to the last quarter of the eleventh or the first decades of the twelfth century CE (S1 Table), thus generally postdating the glasses of groups 3 and 4.

#### Fluxing agents

Judging from the high soda and low potassium concentrations, the alkali source in the Islamic glasses from Vascos was ash from halophytic plants. Plant ash is a complex composite material that contains numerous minor and trace elements that are determined by various factors. Its composition depends on the plant species, the location and soil where it grows, the parts of the plant that are used and the season when the plants are harvested as well as the way the ash is prepared [54–56]. Elements commonly associated with the plant ash component (Na, Mg, P, K, B) and to a lesser degree with the silica source can potentially distinguish different types of plant ash and may reveal differences in the preparation techniques [60, 63–65].

**Fig 6:**
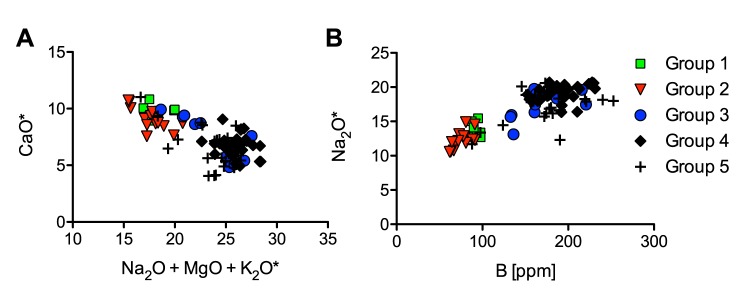
Elements related to the plant ash component in the five different plant ash silica groups. (a) Bivariate plot of CaO against the sum of Na_2_O, MgO and K_2_O; (b) B versus Na_2_O differentiates between types of plant ashes.

It turns out that there is substantial overlap of the silica groups in terms of the plant ash components, except for a cluster consisting of groups 1 and 2. This cluster can be clearly discriminated because of its lower sodium, magnesium and boron contents coupled with the highest lime concentrations (Fig 6). Even though some of the differences can be accounted for by the differences in the quantity of ash used, the ratios of boron to sodium vary sufficiently to deduce that different plant ashes were used in the production of these glasses. It furthermore needs to be borne in mind that a significant fraction of the calcium derives from the silica source, meaning calcium is not a suitable discriminant for the plant ash component alone, unless quartz pebbles were used instead of sand. The glasses of the separate cluster include virtually all fragments considered decorative or architectonic pieces and most of the mould-blown and scratch-engraved glasses. All other plant ash glasses show a wide compositional variability in their plant ash elements with higher sodium, magnesium and boron and overall lower calcium levels.

### Soda-ash lead glass

Six glass fragments from the Vascos assemblage proved to be of soda-ash lead glass with lead concentrations between 40% and 50% and approximately 5% to 7% of soda (S1 Table, Fig 2). These lead glasses have exceptionally high chlorine values (1.7%–2.5%), negatively correlated with calcium. It has been shown that the chlorine content decreases with increasing temperature and calcium oxide content [66]. This could explain the high chlorine level, because the lime concentrations are very low and the melting temperature of glass with high lead content (PbO > 40%) is drastically reduced. Other distinguishing features of these lead glasses are the pronounced levels of arsenic, silver and bismuth that are unlikely associated with the silica or alkali sources (S1 Table), but rather with the lead-bearing component such as galena, litharge or slag [67–68]. Copper, tin and antimony are likewise elevated and it is not clear at this point whether they are remnants of colouring agents or part of the lead source or both. The colours range from colourless and light yellow, greenish and bluish to dark green. All the soda-ash lead glasses date to the tenth and eleventh century and include unguentaria, a bottle, probably the base of a glass lamp and an unusual fragment of an alembic pipe. Islamic lead glasses were first defined by Sayre and Smith [5] as soda silica glasses with contents of lead between 33% and 40%. Many other examples with varying amounts of lead have since been identified in Fustat (Egypt), Timna (Saudi Arabia), the Serçi Limani shipwreck [23], Nishapur [69], Libya [70], Morocco [71] and in numerous sites on the Iberian Peninsula dated to before the thirteenth century CE [21, 59, 72, 73].

## Discussion

### The Vascos glass in its historical context

Ciudad de Vascos was a minor settlement during the late Roman period, located near the Tajo river meadow and a secondary Roman road [11, 74], which may have facilitated the sporadic arrival of glass objects. Our analytical data confirm that the fragments with typological characteristics of Roman objects were all natron glasses, testifying to their survival as residual material in the later Islamic contexts. The natron glasses reflect the import of well-established compositional groups of Levantine and Egyptian origin. In accordance with the numismatic evidence dating to the reigns of the emperors from Constantine to Honorius, the largest group of natron glasses correspond to the HIMT type that is attributed to the fourth and fifth centuries. Consistent with the archaeological evidence is the almost complete lack of early Islamic natron glasses. Only two Islamic Egypt II specimens are attested. This type of glass appears from the beginning of the eighth century and then became, for some time, a dominant glass group in the Near East [19]. The two Egypt II samples thus reflect the latest imports of natron glass into the Iberian Peninsula in the eighth or ninth century CE. Natron glass was subsequently replaced by plant ash glass in Iberia, analogous to the developments in the eastern Mediterranean [19, 22, 42, 75].

Unlike the medieval glass making tradition in central and northern Europe, no potash glass was found in Vascos. Instead, the samples of the tenth and eleventh century correspond to Islamic plant ash glass, testifying to the continuous commercial and technological relationship of Islamic Spain with the eastern Mediterranean. Overall, glass was a rather scarce commodity in Ciudad de Vascos, which is reflected in the quality and quantity of the vitreous materials. Even though the occupation during the late antique period was of much lower density, natron glass makes up a quarter of the entire glass assemblage. This phenomenon might be related to a general decline in the volume of glass that was circulated within the domestic market of Hispania during the early Islamic period, when glass all but disappears from the archaeological record, particularly in more rural areas. A break in the supply and working of glass in Iberia during the early Islamic period is further suggested by an apparent lack of mixed alkali glasses. Whereas both the natron and the plant ash glasses show some signs of recycling in the form of elevated elements usually associated with colouring agents (Co, Cu, Pb, Sb, Sn), no mixing between the two appears to have occurred. The absence of a mixed alkali glass at Vascos is probably due to a decline in the population and near abandonment of the site during the early medieval / early Islamic period and its revival only from the ninth or early tenth century onwards. The renewed occupation was accompanied by the arrival of new glasses made from vegetable soda. The glass was no doubt imported to Vascos as there is no evidence of glass working on site [14]. This has important implications for our understanding of the supply networks and their changes during the Middle Ages.

### The plant ash glass evolution

**Fig 7:**
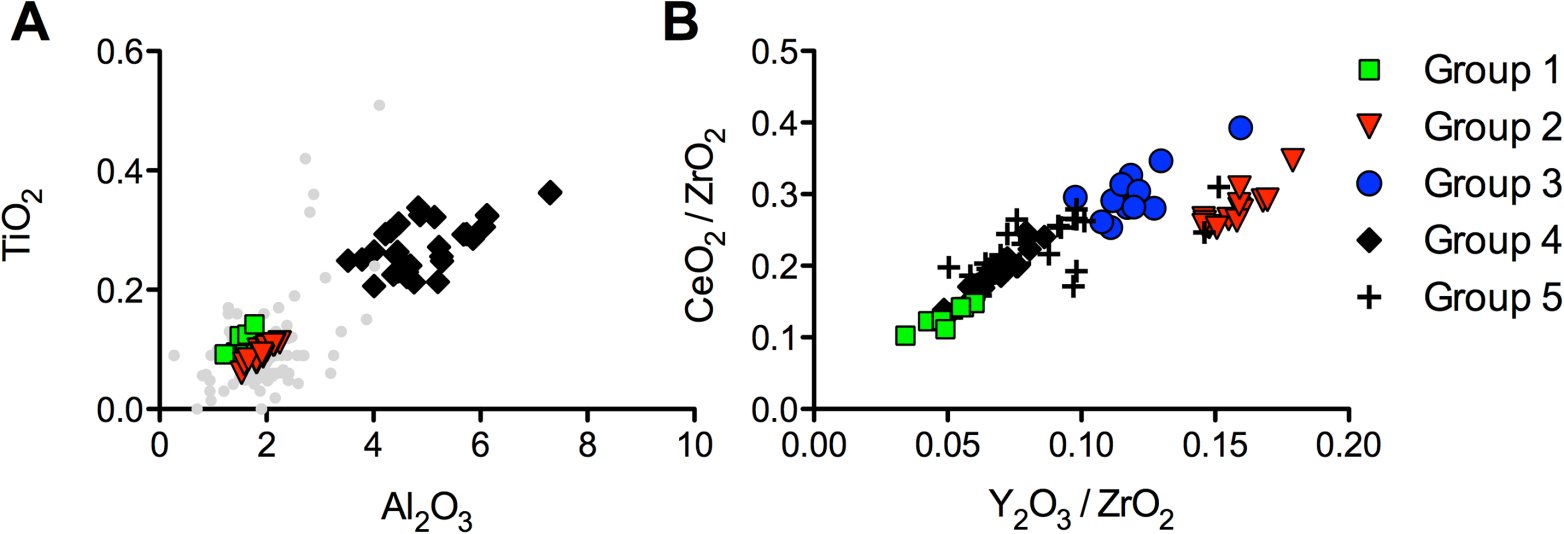
Possible affiliations and provenance of the Vascos plant-ash glass. (a) Aluminium and titanium concentrations in comparison with comparative material from the eastern Mediterranean in light grey from Tyre, Caesarea, and the Serçe Limani shipwreck [23, 76]; (b) distinction of the plant ash groups as a function of yttrium to zirconium and cerium to zirconium ratios.

Our data indicate that the plant ash glasses from Ciudad de Vascos derived from different sources: groups 1, 2 and 3 display lower feldspars and heavy mineral concentrations with distinct zirconium (group 1) and thorium (group 2) signatures, while group 4 was made from a relatively impure silica source with high minor and trace element contaminations. As suggested by our simulation, group 5 may have been the result of mixing between groups 3 and 4 and does therefore not constitute a separate silica source as such. When compared to published data of plant ash glass assemblages from other Mediterranean sites, two major developments become evident: groups 1 and 2 have close similarities with glasses from the eastern Mediterranean both in terms of their typology and composition, whereas no compositional match can be established for group 4. Silica groups 1 and 2 correspond closely to glasses from the early eleventh-century Serçe Limani shipwreck, twelfth-century Caesarea [23], and eleventh-century Tyre [76] as regards the aluminium and titanium levels (Fig 7A). Previously, a correlation has been observed between the geographical origin of glass and the ratios of yttrium to zirconium versus cerium to zirconium [77, 78]. According to these studies, glasses from Egypt typically show low values for both ratios (Y/Zr < 0.08 and Ce/Zr < 0.18), while Syrio-Palestinian glasses have notably higher ratios (Y/Zr > 0.10 and Ce/Zr > 0.24). Even though this comparison works best for natron type glass and caution needs to be exercised when applying it to plant ash glasses, it is noteworthy that group 2 from Vascos meets the criteria for a Levantine provenance as opposed to group 1 that seems more congruent with an Egyptian origin, due to its elevated zirconium (Fig 7B). The data of group 4 (and to a lesser extent group 3), on the other hand, deviate from the Y/Zr versus Ce/Zr ratios typically encountered in eastern Mediterranean glasses [77]. These compositional characteristics strongly suggest that the glasses of plant ash groups 1 and 2 may have been imported from the eastern Mediterranean, whereas group 4 may originate from somewhere else.

In this context, it is important to point out that the fragments of groups 1 and 2 were recovered from areas with the earliest Islamic remains. Group 1 was confined to the affluent dwellings in the surroundings of the kashbah, and the samples of group 2 were found either in the citadel or in the surrounding houses and baths (Fig 1). Even though only one fragment can be securely dated to the mid-ninth to early tenth century (VS105, S1 Table), it seems safe to assume that silica groups 1 and 2 represent the earlier of the plant ash glass compositions from Ciudad de Vascos. Similar compositions were identified in Spain among the eighth- to tenth-century glasses from Gauzon in Asturias (in preparation), tenth-century samples from Cordoba [21] as well as twelfth-century Murcia [59] and Almeria (in preparation). Hence, the analytical and archaeological evidence, together with comparative material suggest that the earliest plant ash groups were imported to Vascos from the eastern Mediterranean, just like Islamic natron glasses (Egypt II) had been imported some years earlier. This indicates that the long-established Mediterranean trade routes were still largely intact as late as the ninth, possibly even the tenth century CE.

### Regional glass production

**Fig 8:**
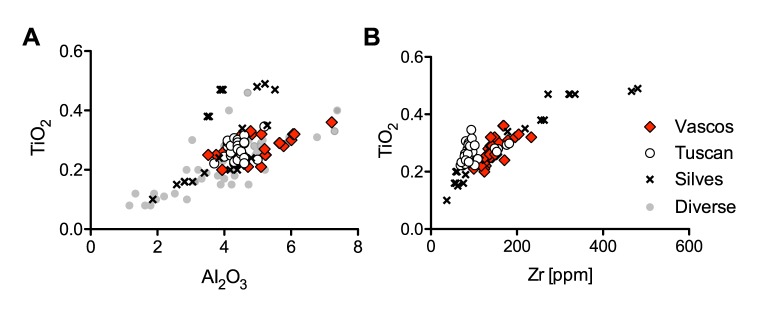
Possible affiliations and provenance of group 4 from Vascos. (a) Aluminium and titanium concentrations in comparison with comparative assemblages from Qasar es-Seghir [23], al-Basra [71], Murcia [59] and Beja [60] in light grey as well as samples from Tuscany [64] and Silves (in preparation); (b) zirconium versus titanium contents highlight differences between Iberian glasses from Vascos and Silves (Portugal) and glasses from various Tuscan sites [64].

No comparative material for group 4 could be identified among published data of contemporary plant ash glasses from the eastern Mediterranean. However, some compositional affinities were found with material from the western Mediterranean. A few isolated examples of a similar composition in terms of high aluminium and titanium contents exist among glasses from Qsar es-Seghir [23], ninth- to tenth-century glass beads from al-Basra [71], as well as Iberian assemblages from Murcia [59] and Silves (in preparation) [79], both dating to the eleventh to thirteenth century, and Beja [60] from the fourteenth and fifteenth centuries (Fig 8A). There is, furthermore, a group of similar glasses from Tuscany and Liguria dating to the thirteenth to sixteenth century [64, 80]. Taking into consideration other trace elements such as zirconium, there is a close correspondence between group 4 and a few samples from Silves and Tuscany, although most of the Tuscan glasses have a different titanium to zirconium ratio (Fig 8B), implying the use of an unrelated silica source.

Unfortunately, no trace element data are available for the other assemblages and only preliminary conclusions can thus be drawn. Nonetheless, there is evidently a higher incidence of this type of soda plant ash glass in Iberia during the eleventh and twelfth centuries than in any other Mediterranean region studied. It may therefore be hypothesised that the glasses of group 4 represent the output of an emerging Iberian glass industry, characterised by high aluminium and high heavy element concentrations as reported for some Spanish sand deposits [81]. When considering the chronology of these developments, almost half the samples of group 4 from Vascos can be attributed with some certainty to the second half of the eleventh or the first decades of the twelfth century, while the vitreous material from Silves (Portugal) and Murcia dates to the twelfth and first half of the thirteenth century [59, 79]. These results suggest that primary plant ash glass production on the Iberian Peninsula started sometime between the tenth and eleventh century CE.

Historical sources about the manufacture or working of glass in medieval Iberia are scarce and notoriously unreliable for the early Middle Ages, but they become more abundant in the second half of the eleventh century [7, 82]. The harvesting of soda-rich halophytic plants in medieval Spain is documented since at least the end of the eleventh century CE [83]. A study of medieval and post-medieval glass from northern Italy found that western Barilla, possibly from Spain, was used alongside Levantine ash in the glass workshops of Altare in the Liguria region already from the thirteenth century onwards [80]. However, Spanish ash (barilla) tends to be significantly richer in potassium than the ash that was used for the Vascos glasses [63]. At the same time, the extensive trade in Syrian plant ash to the western shores of the Mediterranean during the Middle Ages is well documented [84, 85]. For example, Levantine ash was employed in thirteenth- to fourteenth-century Tuscany [63, 64]. The import of plant ash from the eastern Mediterranean to Spain is therefore a possibility, although this must remain tentative as not sufficient evidence is available at this point.

A regional glass production is also indicated by the relative frequency of Islamic soda-ash lead glasses within the Iberian Peninsula. Glasses with compositional characteristics close to the Vascos soda-ash lead glasses were found in Córdoba [21], Murcia [59] as well as Toledo [72], the Baleares [73], Pechina (Almería), Albalat (Cáceres) and Gauzon (in preparation). It has been proposed that these high lead glasses from al-Andalus were made by adding a pure form of lead to either a pre-formed glass or its raw ingredients [21, 59]. A combination of quartz, wood ash and lead oxide was assumed also for wood-ash lead glasses from central European sites [4, 86, 87]. However, given the contamination levels and relative abundance of minor elements such as arsenic, silver and bismuth in the soda-ash lead glasses from Vascos, it appears unlikely that a pure form of lead oxide was used in their production. Instead, the source of lead might have been a slag, the waste product from the lead mining industry flourishing on the Iberian Peninsula since Roman times and well documented in historical sources [88–90].

How the establishment of a Spanish glass industry relates to historical and political developments is not yet clear. The collapse of the Caliphate of Cordoba at the beginning of the eleventh century and the rise of the Taifa Kingdoms most certainly involved a reorganization of the commercial networks. The chemical analysis of glass assemblages can clearly offer valuable new insights into the inter-regional connections and geographical distributions of a high-end product, but there is a need for further investigations of well-defined Iberian glass assemblages.

## Conclusion

The analysis of the glass assemblage from Ciudad de Vascos addresses a lacuna in compositional data of Iberian glasses from the late antique and early Islamic period. The glass compositions reflect the historical evolution of the site and its integration into a wider network of exchange that was subject to political change. We have shown that the pre-Islamic glass encompasses well-established natron-type glass groups that originated in the eastern Mediterranean. The eighth or ninth century saw the import of the last natron glasses in the form of Egypt II, for the first time identified in the archaeological record of the Iberian Peninsula. Subsequently, soda plant ash glass is the principal variant used and it is eventually also produced in Spain. The chronological and the chemical data of Ciudad de Vascos provide the clearest indication yet of an emerging primary plant ash glass production on the Iberian Peninsula. This might date to the first half of the eleventh century, although earlier dates cannot be ruled out at this point. Interestingly, soda-ash lead glass appeared at around the same time as wood ash lead glass in central Europe, possibly in response to the declining availability of natron (or soda ash) from the Levant. The exploitation of waste products from metallurgical activities could have made this technological choice economically viable. Further research is needed to clarify the origin and regional distribution of these technological innovations.

## Supporting information

S1 Table LA-ICP-MS data of the glasses from Ciudad de Vascos. (PDF)

S2 Table LA-ICP-MS data of glass standards in comparison with published values. (PDF)
