# Supplementary material for: Glass import and production in Hispania during the early medieval period: The glass from Ciudad de Vascos (Toledo)
Source: PLoS One. 2017 Jul 26;12(7):e0182129. doi: 10.1371/journal.pone.0182129 (PMC5529010; doi:10.1371/journal.pone.0182129)
Supplement: S1 Table — (PDF) [file pone.0182129.s001.pdf]

S1 Table: LA-ICP-MS data of the glass from Ciudad de Vascos

						wt%											
	Sample	REF	Context date	Colour	Type	Na_2O	MgO	Al_2O_3	SiO_2	P_2O_5	Cl	K_2O	CaO	TiO_2	MnO	Fe_2O_3	PbO
**Natron**																	
**Levantine**	VS008	8/VS85/C-10/BAÑO DE LA MORA/s.UE	Superficial	light green	bottle neck body fragment	16.8	1.03	2.77	66.5	0.14	0.83	0.97	7.68	0.14	0.95	1.04	0.61
	VS046	49/VS96/Oeste Barbacana/2	2nd half 11th - 1st 1/4 12th c.	bluish	wide bottle rim	16.0	0.98	2.83	67.0	0.15	0.81	0.96	8.31	0.15	0.68	1.06	0.62
	VS050	54/VS96/0-5/W Alcazaba/2	10th - 11th c.	bluish	undetermined body fragment	15.8	0.48	2.70	70.1	0.14	1.01	0.67	8.02	0.05	0.31	0.50	0.01
	VS051	55/VS96/0-5/6/8	11th c.	colourless	undetermined body fragment	16.5	0.59	2.68	69.7	0.17	1.00	0.74	7.18	0.08	0.47	0.52	0.02
	VS074	85/VS98/0-5/15G/NS	2nd half 11th c.	bluish	dish rim	15.4	0.95	2.66	69.0	0.17	0.82	0.88	9.01	0.10	0.24	0.60	0.00
	VS084	95/VS98/0-5A/3/4	2nd half 11th c.	bluish	dish rim	15.4	1.02	3.01	67.8	0.18	0.74	1.00	9.34	0.11	0.45	0.79	0.01
	VS087	98/VS98/0-5/15E/128	11th c.	colourless	undetermined body fragment	14.6	0.57	2.87	71.0	0.11	1.02	0.96	8.34	0.06	0.02	0.33	0.00
	VS096	110/VS001/0-5/E2/NS	Superficial	colourless	dish rim	15.8	0.84	2.83	68.9	0.13	0.79	0.73	8.06	0.11	0.54	0.77	0.12
	VS104	118/VS001/0-5/B9/580	2nd half 11th - 1st 1/4 12th c.	bluish	undetermined body fragment	15.6	0.54	2.65	71.1	0.12	1.02	0.54	7.78	0.06	0.10	0.37	0.00
	VS106	121/VS05/0-4/Calle/93C/380/nº1	2nd half 11th c.	greenish	dish rim	16.0	0.58	3.26	68.9	0.17	0.85	0.71	8.79	0.07	0.02	0.45	
	VS127	150/VS08/PORTILLO III/NS	Superficial	bluish	undetermined handle	14.7	0.47	2.75	72.3	0.09	1.02	0.44	7.68	0.05	0.10	0.33	0.00
	VS128	151/VS08/PUERTA W/NS	Superficial	bluish	goblet shaft	14.6	0.83	2.95	70.0	0.10	0.82	0.93	8.57	0.10	0.20	0.61	0.04
	VS152	171/VS02/0-5/Rampa/3	Late Roman	light green	undetermined body fragment	15.7	0.52	2.64	71.1	0.08	1.10	0.85	7.49	0.06	0.02	0.32	0.00
**Roman Sb**	VS057	61/VS97/0-5/10/16 Bajo tejas	2nd half 11th c.	colourless	bowl rim	18.3	0.42	2.01	71.3	0.03	1.18	0.43	5.30	0.06	0.02	0.32	0.00
	VS097	111/VS001/0-5/C9/587	10th c.	amber	undetermined handle	16.5	0.34	1.80	72.7	0.15	1.17	0.50	6.36	0.05	0.04	0.28	0.00
**Egypt II**	VS018	20/ VS87/0-1/ UE 218	Before 11th c. (8th - 9th ?)	colourless	bottle rim	13.3	0.50	2.51	69.1	0.07	0.93	0.36	11.2	0.25	0.75	0.91	0.00
	VS031	33/VS93/0-1/11/6	10th - 11th c.	bluish	flat glass	14.6	0.45	2.30	69.6	0.07	1.20	0.33	10.3	0.23	0.02	0.79	0.00
**Foy-2**	VS035	38/VS94/0-1/6/52	10th - 11th c.	bluish	bottle base ?	18.3	1.27	2.25	66.7	0.03	1.26	0.37	7.19	0.12	1.51	0.71	0.00
	VS045	48/VS96/0-5/6/10	Before 11th c.	greenish	pitcher rim	19.1	0.88	2.15	67.4	0.03	1.22	0.34	6.23	0.13	1.46	0.82	0.00
	VS062	69/VS98/0-5/10/72	2nd half 11th - 1st 1/4 12th c.	greenish	undetermined rim	18.8	0.98	2.68	66.7	0.05	1.10	0.46	6.36	0.24	1.43	0.98	0.00
**HIMT / Foy-1**	VS001	1/VS77/0-1/E	late Roman - 10th - 11th c.	greenish	goblet base	20.7	1.39	3.18	63.4	0.04	1.23	0.33	5.00	0.45	2.13	1.90	0.00
	VS017	19/VS87/0-1/UE 7	10th - 11th c.	greenish	flat glass	18.5	1.22	2.73	66.1	0.05	0.98	0.37	6.00	0.43	1.77	1.62	0.00
	VS030	32/VS93/0-1/11/6	10th - 11th c.	greenish	undetermined body fragment	19.9	1.37	2.94	64.6	0.04	1.27	0.34	4.90	0.46	2.06	1.89	0.00
	VS034	37/VS94/0-1/26/2	10th - 11th c.	amber	dish rim	18.2	1.03	3.05	64.1	0.14	0.83	0.62	6.76	0.35	1.82	2.93	0.00
	VS036	39/VS95/0-1/34/1	2nd half 11th c.	greenish	dish rim	16.5	0.94	3.12	68.4	0.08	0.92	0.42	4.99	0.35	1.81	2.34	0.00
	VS042	45/VS96/0-5/10/1	2nd half 11th c.	greenish	bottle base	16.8	1.07	3.02	65.0	0.21	0.90	0.43	5.12	0.61	1.71	4.93	0.00
	VS060	64/VS97/0-5/Alc/ 2	2nd half 11th c.	greenish	flat glass	18.2	1.07	2.79	64.8	0.08	0.93	0.51	6.17	0.51	2.39	2.16	0.01
	VS072	83/VS98/0-5/13E/146	2nd half 11th - 1st 1/4 12th c.	greenish	dish rim	15.5	1.07	2.78	69.3	0.06	0.95	0.36	5.97	0.30	1.63	1.88	0.00
	VS075	86/VS98/NS	Superficial	amber	dish rim	19.0	1.13	2.59	63.9	0.10	1.11	0.41	5.88	0.46	1.89	3.27	0.00
	VS083	94/VS98/0-5A/3/3	2nd half 11th c.	greenish	goblet rim	19.3	1.02	2.70	65.4	0.05	0.99	0.44	6.14	0.43	1.85	1.45	0.00
	VS086	97/VS98/0-5/13H/167	2nd half 11th - 1st 1/4 12th c.	amber	flat glass	18.0	0.86	2.95	65.3	0.16	0.96	0.54	5.61	0.52	1.15	3.75	0.00
	VS100	114/VS001/0-5/E2/NS	Superficial	light green	undetermined rim	16.7	0.75	2.65	68.8	0.08	0.82	0.41	5.07	0.57	1.49	2.42	0.00
	VS101	115/VS001/0-5/C7/413	Late Roman - 10th c.	greenish	undetermined body fragment	16.7	0.88	3.24	68.6	0.10	0.84	0.50	4.65	0.39	1.10	2.80	0.00
	VS107	122/VS05/0-4/Calle/92B/384/nº384	2nd half 11th c.	greenish	dish rim	17.9	0.90	2.72	67.0	0.03	0.93	0.33	5.96	0.47	1.72	1.79	0.00
	VS126	149/VS08/PUERTA W/5	10th - 11th c.	greenish	goblet base	17.6	0.88	2.95	67.2	0.03	1.05	0.36	4.78	0.58	2.28	2.02	0.01
	VS129	82/VS98/0-5/13I/162	2nd half 11th c.	greenish	undetermined body fragment	19.4	0.96	2.91	65.1	0.03	1.07	0.40	5.55	0.71	1.61	2.05	0.00
	VS151	170/VS98/0-5/18E/153	2nd half 11th c.	amber	undetermined body fragment	17.1	1.07	2.47	65.0	0.20	0.87	0.42	5.93	0.41	2.45	3.85	0.01
**Soda-rich plant ash**																	
**Group 1**	VS022	24/VS90/0-1/A-1/3/16	10th - 11th c.	bluish	decorative cut flat glass	14.8	2.10	1.41	65.7	0.47	0.73	2.79	9.80	0.09	1.02	0.75	0.10
	VS023	25/VS91/0-1/5/UE 9	10th - 11th c.	colourless	decorative cut flat glass	12.6	2.17	1.58	67.2	0.53	0.67	2.52	10.7	0.10	0.27	0.85	0.62
	VS033	35/VS93/0-1/12/UE 14	10th - 11th c.	colourless	decorative cut flat glass	12.5	2.02	1.19	68.7	0.55	0.89	2.67	8.77	0.09	1.59	0.72	0.02
	VS038	41/VS95/0-1/33/11	2nd half 11th c.	bluish	decorative cut flat glass	13.9	2.18	1.77	66.9	0.37	0.82	2.05	9.69	0.11	0.95	0.82	0.27
	VS040	43/VS95/0-1/36/13	2nd half 11th c.	colourless	decorative cut flat glass	14.9	2.28	1.45	63.2	0.72	1.16	2.01	9.48	0.12	2.94	0.91	0.56
	VS145	163/VS03/0-4/Patio/R13/140/nº19	Before 11th c. (8th - 9th ?)	colourless	undetermined body fragment	13.2	2.30	1.62	68.2	0.39	0.98	1.77	9.22	0.12	1.18	0.73	0.09
	VS146	164/VS06/0-4/Calle Sur/91D/NS/nº21	10th - 11th c.	colourless	flat glass	12.8	2.14	1.75	68.1	0.43	0.76	1.77	9.91	0.14	0.72	0.82	0.50
	VS005	5/VS81/UE 1/¿baños?	Superficial	colourless	undetermined body fragment	12.5	3.21	1.80	68.5	0.30	0.87	2.32	8.90	0.08	0.97	0.45	0.00
	VS006	6/VS81/C-5/nivel entre tejas/ ¿baños?	10th - 11th c.	colourless	undetermined body fragment	12.5	3.22	1.79	68.4	0.30	0.87	2.32	8.99	0.08	0.88	0.45	0.00
	VS014	16/VS86-87/0-1/s.UE	Superficial	colourless	tubular unguentaria base	11.5	2.28	1.94	68.9	0.44	0.97	3.06	9.00	0.10	1.10	0.57	0.00
	VS019	21/VS88/0-1/REC 1/UE 39	10th - 11th c.	colourless	engraved bottle body fragment	12.2	2.86	2.23	67.9	0.39	0.98	2.46	9.26	0.11	0.77	0.70	0.00
	VS020	22/VS89/0-2/UE 2125	10th - 11th c.	colourless	undetermined mould blown body fragment	14.3	2.69	1.82	67.5	0.34	1.05	2.59	7.54	0.10	1.32	0.60	0.00

	ppm																																												
Sample	Li	B	V	Cr	Co	Ni	Cu	Zn	Ga	As	Rb	Sr	Y	Zr	Nb	Mo	Ag	Cd	In	Sn	Sb	Cs	Ba	La	Ce	Pr	Nd	Sm	Eu	Gd	Tb	Dy	Ho	Er	Tm	Yb	Lu	Hf	Ta	W	Pt	Au	Bi	Th	U
**Natron**																																													
VS008	27.8	152	24.6	22.5	21.7	15.2	880	89.5	4.42	4.73	13.8	516	7.33	77.0	2.31	2.59	1.78	0.09	0.00	829	760	0.26	322	7.29	12.3	1.61	6.84	1.35	0.42	1.56	0.25	1.19	0.26	0.75	0.10	0.72	0.10	1.84	0.14	0.57	0.01	0.23	0.61	1.23	1.01
VS046	20.1	137	22.3	24.1	20.2	12.7	922	102	4.42	8.25	13.2	450	7.25	84.9	2.43	1.89	7.70	0.09	0.00	175	694	0.35	302	7.68	13.3	1.71	7.04	1.45	0.40	1.51	0.23	1.23	0.26	0.71	0.10	0.71	0.10	2.03	0.15	0.40	0.00	0.15	0.59	1.41	1.07
VS050	3.31	136	6.69	13.1	31.4	5.24	122	14.9	3.50	1.81	8.91	406	6.47	34.1	1.14	0.72	0.26	0.03	0.02	11.6	129	0.12	270	5.81	10.2	1.30	5.58	1.14	0.37	1.23	0.19	1.04	0.21	0.60	0.08	0.57	0.08	0.83	0.07	0.03	0.00	0.01	0.01	0.80	1.05
VS051	5.31	151	13.3	16.5	14.4	8.60	527	25.5	3.48	3.73	9.86	388	6.39	49.8	1.55	1.34	0.35	0.05	0.00	59.3	833	0.15	218	6.21	11.0	1.37	5.88	1.18	0.37	1.25	0.19	1.05	0.22	0.63	0.08	0.60	0.09	1.24	0.10	0.10	0.00	0.01	0.13	1.08	0.91
VS074	4.78	107	15.3	18.5	11.6	9.07	14.8	14.8	3.51	1.26	6.87	484	6.29	54.4	1.72	1.08	0.08	0.03	0.00	1.22	1.57	0.08	246	6.27	11.3	1.39	5.71	1.12	0.35	1.07	0.18	1.04	0.22	0.60	0.08	0.58	0.09	1.34	0.10				0.01	0.96	0.91
VS084	15.1	108	19.5	19.1	14.5	13.4	23.3	24.4	4.23	2.48	11.5	548	7.50	56.4	1.98	1.80	0.10	0.03	0.01	3.79	18.4	0.18	300	7.78	13.3	1.71	6.99	1.41	0.42	1.32	0.21	1.26	0.26	0.73	0.10	0.67	0.10	1.37	0.12	0.15	0.00	0.00	0.01	1.17	1.12
VS087	3.35	106	6.99	12.1	1.06	3.41	4.91	6.74	3.66	1.81	12.9	423	6.90	36.9	1.25	1.09	0.05	0.02	0.00	0.32		0.12	213	6.45	11.8	1.44	6.05	1.22	0.39	1.14	0.20	1.12	0.23	0.64	0.08	0.58	0.08	0.91	0.07	0.06		0.00	0.00	0.82	0.52
VS096	10.1	120	18.0	19.6	15.6	9.99	401	72.1	4.07	5.70	10.6	464	7.09	59.2	1.95	1.42	0.68	0.07	0.00	158	704	0.22	281	7.46	13.3	1.65	6.75	1.37	0.40	1.22	0.21	1.19	0.25	0.68	0.09	0.63	0.09	1.45	0.11	0.28	0.00	0.07	0.35	1.17	1.00
VS104	3.34	142	9.43	7.12	3.26	4.36	6.54	9.33	3.13	0.76	6.12	398	6.21	33.5	1.17	0.43	0.09	0.02	0.01	0.47	1.26	0.05	181	5.45	9.84	1.25	5.40	1.11	0.36	1.28	0.19	0.99	0.21	0.60	0.08	0.56	0.08	0.84	0.07	0.04	0.00		0.00	0.73	0.96
VS106	3.30	90.4	9.72		1.32	4.85	4.00	8.36	3.75		8.93	402	6.49	39.1	1.42	0.44	0.09	0.03	0.00	0.24		0.04	238	5.76	10.3	1.34	5.61	1.15	0.39	1.30	0.19	0.98	0.22	0.60	0.08	0.55	0.09	0.99	0.08	0.06	0.00			0.76	0.78
VS127	2.76	83.6	6.19		1.71	5.99	3.25	9.22	3.55	0.86	6.40	389	6.55	34.5	1.14	0.27	1.59	0.04	0.00	0.49	0.27	0.07	217	6.52	11.5	1.44	5.84	1.20	0.36	1.02	0.18	1.05	0.23	0.60	0.08	0.53	0.09	0.86	0.07	0.14	0.00	0.16	0.02	0.90	1.02
VS128	41.6	80.9	12.3	1.31	6.61	5.88	208	32.0	4.04	3.37	23.2	466	7.08	55.3	1.92	1.00	0.45	0.06	0.01	38.8	494	0.51	249	7.71	14.1	1.67	6.85	1.37	0.39	1.16	0.21	1.18	0.24	0.68	0.09	0.59	0.09	1.38	0.11	0.15	0.00	0.47	0.08	1.21	0.97
VS152	3.08	109	6.99		1.03	3.08	6.22	6.58	3.56	1.06	11.5	401	6.70	39.4	1.27	0.72	0.04	0.03	0.00	0.45		0.08	195	6.79	12.5	1.44	5.89	1.21	0.37	0.89	0.18	1.05	0.22	0.59	0.08	0.52	0.08	1.02	0.07	0.05	0.00	0.00		0.85	0.49
VS057	3.11	223	5.90	10.0	1.04	2.79	8.61	19.1	2.59	24.8	5.71	304	4.76	41.6	1.19	0.09	0.14	0.11	0.00	0.34	4429	0.07	136	4.98	8.67	1.10	4.61	0.91	0.27	0.91	0.14	0.80	0.16	0.46	0.06	0.45	0.07	1.04	0.07	0.04	0.00	0.02	0.03	0.79	0.83
VS097	2.93	311	6.11	10.4	1.26	2.92	3.87	7.94	2.49	1.15	5.28	298	5.25	36.0	0.97	0.25	0.05	0.02	0.00	0.51	1.33	0.04	156	5.34	9.10	1.16	4.79	1.00	0.29	0.90	0.15	0.85	0.18	0.51	0.06	0.43	0.07	0.91	0.06	0.03	0.00	0.00	0.00	0.72	0.51
VS018	4.01	39.0	19.8	25.0	3.67	7.60	6.06	35.0	4.22	1.98	5.33	174	6.91	170	3.76	0.26	0.27	0.12	0.01	0.42		0.07	225	6.84	12.9	1.59	6.35	1.31	0.35	1.34	0.22	1.11	0.24	0.70	0.10	0.74	0.12	3.93	0.21	0.10	0.01		0.01	1.50	1.28
VS031	3.32	37.5	17.7	25.1	2.37	5.64	2.74	11.9	3.46	1.15	4.91	157	6.09	155	3.26	0.10	0.26	0.08	0.01	0.36	0.03	0.06	147	6.76	12.6	1.48	5.97	1.18	0.33	1.06	0.19	1.03	0.22	0.62	0.09	0.64	0.10	3.52	0.19	0.06	0.00	0.00	0.01	1.43	0.93
VS035	5.22	225	28.0	21.1	6.96	10.2	35.9	18.4	4.08	2.30	4.00	547	6.57	53.9	1.99	4.39	0.17	0.05	0.00	2.41	4.87	0.04	374	6.51	10.8	1.43	6.05	1.21	0.38	1.39	0.21	1.09	0.23	0.67	0.09	0.64	0.09	1.33	0.11	0.14	0.00	0.00	0.01	0.91	0.78
VS045	3.88	187	25.6	20.3	11.0	11.1	34.7	17.8	4.01	2.18	4.64	457	7.10	66.7	2.04	3.41	0.11	0.01	0.00	0.54		0.04	181	7.08	10.4	1.52	6.34	1.33	0.38	1.43	0.22	1.14	0.24	0.71	0.10	0.68	0.11	1.64	0.12	0.16	0.00		0.00	0.91	0.85
VS062	5.28	182	28.1	32.5	7.68	11.2	96.1	27.0	4.64	2.82	6.11	449	7.71	122	3.03	3.58	0.29	0.04	0.01	3.24	13.2	0.07	360	7.56	13.4	1.69	7.12	1.52	0.42	1.43	0.24	1.31	0.27	0.79	0.11	0.78	0.12	2.78	0.18	0.23	0.00	0.01	0.01	1.33	0.90
VS001	5.48	228	57.8	60.0	12.6	16.4	59.1	30.0	6.22		4.28	386	9.50	216	5.10	7.34	0.60	0.06	0.02	0.64		0.05	707	9.01	16.2	2.03	8.71	1.82	0.61	2.10	0.33	1.65	0.37	1.06	0.16	1.09	0.16	4.81	0.30	0.31	0.01		0.00	1.85	1.19
VS017	5.24	200	43.1	57.4	8.28	18.0	38.7	29.7	5.52	4.43	4.94	474	9.88	246	5.06	3.73	0.45	0.11	0.02	2.78	2.37	0.04	217	10.5	16.7	2.31	9.54	1.93	0.52	2.03	0.33	1.67	0.37	1.06	0.15	1.06	0.18	5.64	0.30	0.25	0.01		0.03	2.17	1.62
VS030	4.99	218	58.8	64.6	12.7	16.5	59.6	33.5	6.28	4.40	6.53	386	9.80	227	5.39	7.29	0.38	0.08	0.02	0.75	0.36	0.09	707	10.3	18.8	2.28	9.36	1.85	0.53	1.75	0.31	1.66	0.36	1.07	0.16	1.07	0.16	5.06	0.32	0.35	0.01	0.00	0.02	2.08	1.32
VS034	7.59	195	94.3	57.0	11.6	25.2	48.5	39.1	5.84	13.9	6.80	551	11.1	178	4.24	3.39	0.33	0.09	0.02	1.16		0.09	274	11.2	15.3	2.53	10.6	2.26	0.58	2.43	0.37	1.96	0.41	1.21	0.15	1.17	0.17	3.96	0.24	0.27	0.00	0.00	0.02	1.69	2.00
VS036	4.01	190	77.6	58.2	10.5	21.1	41.5	31.1	5.89	8.98	6.55	391	10.2	168	4.10	2.84	0.30	0.11	0.02	0.84		0.07	299	9.74	14.0	2.21	9.33	2.04	0.55	2.16	0.33	1.72	0.38	1.10	0.15	1.00	0.15	3.82	0.23	0.27	0.01	0.00	0.02	1.52	1.57
VS042	4.58	131	114	86.3	15.1	46.2	96.9	54.5	6.77	23.6	6.59	368	16.0	303	6.36	5.24	0.48	0.06	0.04	0.72		0.11	188	15.8	18.0	3.59	15.1	3.15	0.82	3.52	0.53	2.81	0.59	1.68	0.22	1.68	0.25	6.84	0.37	0.34	0.01	0.00	0.03	2.24	1.65
VS060	5.20	132	48.5	85.5	16.4	14.4	108	41.0	6.52	5.96	5.36	491	10.7	275	5.66	2.69	0.57	0.06	0.02	15.8	7.95	0.08	1338	10.2	22.9	2.36	9.71	1.97	0.61	2.10	0.32	1.83	0.38	1.12	0.16	1.16	0.19	6.19	0.32	0.71	0.01	0.01	0.04	2.21	1.25
VS072	4.93	164	78.9	39.8	14.4	20.2	34.5	27.6	5.28	5.97	5.36	467	9.83	146	3.91	2.92	0.17	0.08	0.02	1.10		0.07	318	10.2	15.4	2.24	9.24	1.85	0.52	1.73	0.29	1.62	0.34	0.96	0.14	0.93	0.14	3.32	0.21				0.01	1.56	0.89
VS075	4.76	173	86.4	64.7	12.5	24.9	69.3	42.5	5.73	15.7	5.92	412	13.2	263	5.21	2.54	0.32	0.04	0.02	0.99		0.08	270	13.8	17.5	3.04	12.6	2.52	0.65	2.44	0.41	2.32	0.49	1.39	0.19	1.37	0.21	5.77	0.30				0.03	2.20	1.28
VS083	5.38	181	41.3	57.7	9.79	15.3	75.9	33.8	5.43	4.07	5.86	452	9.68	244	4.90	5.00	0.35	0.06	0.01	2.82	7.08	0.06	360	9.91	17.4	2.21	8.97	1.79	0.48	1.67	0.28	1.63	0.35	0.98	0.14	1.01	0.16	5.52	0.28	0.32	0.01	0.00	0.02	2.16	1.60
VS086	4.70	47.6	131	71.0	10.8	25.9	63.5	39.8	6.15	18.7	7.64	415	14.6	260	5.78	2.06	0.31	0.06	0.03	0.84	0.32	0.11	196	15.6	18.7	3.45	14.0	2.94	0.75	2.73	0.47	2.61	0.54	1.53	0.22	1.50	0.23	5.73	0.33	0.29	0.00	0.00	0.04	2.14	1.04
VS100	3.87	93.1	69.5	76.7	10.1	23.3	37.2	31.5	5.48	8.91	6.26	371	12.1	300	5.96	3.86	0.38	0.07	0.03	0.67	0.34	0.07	224	12.3	17.9	2.69	11.1	2.33	0.58	2.04	0.36	2.11	0.45	1.22	0.18	1.28	0.20	6.70	0.34	0.32	0.00	0.00	0.02	2.32	1.23
VS101	4.61	133	70.9	54.3	9.79	28.8	47.6	36.4	5.72	12.4	7.58	347	12.2	190	4.48	3.18	0.23	0.02	0.03	0.61	0.39	0.11	215	13.2	16.9	2.88	12.0	2.49	0.66	2.19	0.39	2.16	0.45	1.26	0.17	1.22	0.18	4.31	0.26	0.21	0.00	0.00	0.03	1.80	1.73
VS107	3.15	186	51.6		9.78	15.8	52.9	32.9	5.91		4.72	441	9.71	244	5.01	3.04	0.55	0.10	0.01	1.97		0.02	824	9.11	16.1	2.11	8.91	1.80	0.56	2.11	0.31	1.66	0.34	1.00	0.14	1.06	0.15	5.64	0.30	0.32	0.01	0.01		1.97	1.30
VS126	3.13	249	57.7	26.5	12.7	18.1	65.3	43.1	6.33	2.94	4.97	364	10.7	274	6.14	5.44	0.39	0.11	0.01	7.79		0.05	816	10.9	19.4	2.42	9.68	2.01	0.57	1.71	0.30	1.76	0.41	1.17	0.17	1.16	0.19	6.16	0.34	0.42	0.01	0.00	0.01	2.32	1.47
VS129	3.25	76.9	65.8	61.2	12.0	14.4	33.7	30.8	6.13	2.92	5.62	418	11.9	377	7.16	4.62	0.66	0.10	0.01	0.64		0.07	765	11.9	21.8	2.62	10.6	2.26	0.59	1.86	0.35	1.99	0.44	1.24	0.19	1.33	0.22	8.38	0.42	0.36	0.01	0.00	0.00	2.86	1.43
VS151	5.13	191	104	20.6	21.4	36.8	98.0	58.0	6.12	25.5	5.68	471	13.8	212	4.98	5.09	0.32	0.11	0.03	10.6	0.41	0.07	305	15.6	18.7	3.20	13.2	2.65	0.67	2.09	0.40	2.34	0.50	1.38	0.21	1.31	0.20	4.77	0.28	0.49	0.01	0.01	0.03	2.02	1.82
**Soda-rich plant ash**																																													
VS022	55.7	87.5	10.5	13.4	5.96	6.73	170	42.5	3.30	1.59	9.96	440	5.42	84.7	1.86	2.30	0.29	0.10	0.00	10.7	1.72	0.16	261	7.22	13.8	1.62	6.53	1.21	0.30	1.23	0.19	0.91	0.18	0.54	0.07	0.51	0.08	2.05	0.12	0.09	0.00		0.05	1.51	0.70
VS023	81.7	83.6	9.96	14.5	2.63	5.81	427	59.3	3.21	3.66	9.85	360	5.55	109	2.10	0.92	0.29	0.08	0.00	56.6	0.38	0.19	129	7.71	14.9	1.67	6.66	1.26	0.29	1.25	0.18	0.90	0.20	0.57	0.07	0.53	0.09	2.52	0.14	0.06	0.00		0.06	1.74	0.67
VS033	46.4	98.0	18.8	10.8	7.45	14.9	400	49.1	3.77	2.70	8.77	536	5.68	95.1	1.80	4.62	0.31	0.13	0.00	12.0	1.26	0.15	595	7.51	14.9	1.65	6.66	1.24	0.33	1.14	0.18	0.94	0.19	0.55	0.08	0.50	0.08	2.24	0.11	0.38	0.00	0.01	0.04	1.53	0.88
VS038	70.4	89.8	12.4	16.1	8.43	6.30	189	45.6	3.61	1.94	9.51	393	5.24	89.7	2.30	1.71	0.26	0.11	0.00	36.2		0.18	215	7.26	14.0	1.62	6.41	1.28	0.28	1.31	0.19	0.92	0.19	0.53	0.08	0.51	0.08	2.18	0.14	0.09	0.00	0.00	0.04	1.64	0.86
VS040	20.9	95.0	26.5	17.9	11.2	25.6	425	105	4.66	4.09	6.93	677	6.29	120	2.20	10.4	0.44	0.18	0.01	54.9		0.09	203	7.89	14.7	1.73	6.91	1.34	0.28	1.39	0.20	0.98	0.20	0.57	0.08	0.60	0.10	2.84	0.15	0.16	0.00	0.01	0.12	1.72	1.01
VS145	78.2	97.6	10.2		5.65	4.80	91.7	44.8	3.96	1.46	7.92	379	6.16	136	2.49	1.70	0.21	0.12	0.00	10.1		0.15	170	9.63	18.3	2.01	7.85	1.40	0.29	1.07	0.18	0.99	0.21	0.59	0.09	0.59	0.10	3.19	0.17	0.08	0.00	0.00	0.01	2.20	1.13
VS146	54.3	86.3	15.1		5.00	8.39	296	79.7	3.94	3.56	10.7	374	6.61	182	2.98	1.96	0.38	0.13	0.00	44.2		0.19	244	10.9	20.5	2.22	8.52	1.54	0.34	1.19	0.21	1.15	0.24	0.64	0.09	0.65	0.11	4.16	0.19	0.18	0.00	0.01	0.05	2.48	1.13
VS005	5.47	79.1	11.4	9.98	3.00	7.57	33.2	22.1	3.46		13.7	437	5.83	34.4	1.49	2.35	0.08	0.04	0.00	0.37		0.14	217	5.65	10.6	1.37	5.95	1.25	0.37	1.33	0.21	1.00	0.21	0.56	0.07	0.49	0.07	0.86	0.09	0.31	0.00		0.01	0.74	0.36
VS006	5.48	79.0	11.1	9.36	2.88	7.49	30.4	21.8	3.35		13.7	440	5.77	33.9	1.49	2.18	0.10	0.03	0.00	0.35		0.13	210	5.60	10.3	1.33	5.82	1.22	0.36	1.30	0.21	1.00	0.19	0.57	0.07	0.49	0.08	0.85	0.08	0.29	0.00		0.01	0.72	0.34
VS014	5.21	68.7	14.2	15.6	3.51	8.47	35.2	26.8	3.86	2.52	11.6	689	6.25	40.3	1.80	2.51	0.13	0.04	0.00	1.05	0.20	0.10	224	6.29	11.8	1.47	6.41	1.34	0.37	1.32	0.23	1.03	0.21	0.60	0.08	0.53	0.08	1.01	0.10	0.32	0.00		0.02	0.86	0.45
VS019	6.46	91.4	17.4	17.5	3.35	11.9	23.9	37.8	3.95	2.34	13.6	649	6.83	41.4	2.12	1.69	0.03	0.05	0.00	0.45		0.14	220	6.87	12.1	1.58	6.66	1.32	0.39	1.33	0.22	1.11	0.22	0.64	0.09	0.57	0.09	0.96	0.12	0.20	0.00		0.02	1.03	0.70
VS020	5.70	91.1	14.8	15.2	3.84	9.43	42.8	27.2	3.89	2.63	13.6	485	7.07	39.6	1.79	2.89	0.07	0.03	0.01	0.61	0.02	0.14	204	6.72	12.7	1.57	6.67	1.42	0.40	1.33	0.23	1.15	0.22	0.65	0.09	0.60	0.08	0.95	0.10	0.34	0.00		0.02	0.93	0.43

Group	Sample	REF	Context date	Colour	Type	wt%											
	Sample	REF	Context date	Colour	Type	Na_2O	MgO	Al_2O_3	SiO_2	P_2O_5	Cl	K_2O	CaO	TiO_2	MnO	Fe_2O_3	PbO
Group 2	VS028	30/VS93/0-1/15/UE 2	2nd half 11th c.	colourless	undetermined base	12.3	3.13	1.52	69.5	0.28	0.81	2.55	8.60	0.06	0.83	0.32	0.00
	VS066	75/VS98/0-5/10/16 Bajo tejas	11th c.	colourless	cut, decorative piece	11.7	2.96	1.98	70.4	0.35	0.88	2.36	7.47	0.10	1.05	0.56	0.00
	VS076	87/VS98/0-5/13D/NS	Superficial	colourless	undetermined body fragment	10.6	2.17	1.99	69.5	0.40	0.82	2.68	9.94	0.10	0.95	0.62	0.00
	VS085	96/VS98/0-5/13F/146	2nd half 11th - 1st 1/4 12th c.	colourless	undetermined body fragment	10.5	2.32	2.12	68.8	0.41	0.88	2.57	10.5	0.11	0.99	0.64	0.00
	VS090	101/VS99/0-5/12E/184	2nd half 11th c.	greenish	bottle rim	10.4	2.29	2.10	68.8	0.40	0.85	2.56	10.6	0.11	1.06	0.65	0.00
	VS105	120/VS02/0-5/F3/823	9th - 10th c.	colourless	undetermined body fragment	13.0	2.74	1.79	68.2	0.30	0.88	2.96	8.36	0.08	1.09	0.45	0.00
	VS111	134/VS07/PUERTA S/Ext/Recinto Puerta/1	Superficial	greenish	decorative rim	12.7	3.28	1.92	68.4	0.28	0.84	2.15	8.64	0.09	1.09	0.51	0.00
	VS130	152/VS08/PUERTA S/36	2nd half 11th c.	colourless	bottle base	14.7	3.25	1.57	66.5	0.27	0.90	2.56	8.62	0.08	1.00	0.43	0.00
	VS144	162/VS06/0-4/Calle Sur/91C/481/nº16	11th c.	colourless	undetermined body fragment	12.0	3.35	1.64	68.1	0.33	0.88	2.21	9.59	0.08	1.20	0.46	0.00
	VS147	165/VS03/0-4/R12/113/nº15	11th c.	colourless	undetermined body fragment	11.9	3.00	1.89	69.4	0.32	0.91	2.18	8.43	0.09	1.25	0.52	0.00
Group 3	VS016	18/VS87/0-1/UE 87	2nd half 11th c.	bluish	tubular unguentaria base	15.9	3.57	1.74	65.6	0.44	1.09	1.27	9.26	0.08	0.12	0.67	0.05
	VS026	28/VS93/0-1/11/UE 16	10th - 11th c.	greenish	unguentaria base and body	15.3	3.25	2.23	63.1	0.52	0.96	1.88	9.17	0.11	1.98	0.99	0.24
	VS039	42/VS95/0-1/6/100	10th - 11th c.	greenish	undetermined base / unguentaria ?	13.0	4.03	2.62	65.6	0.57	1.00	1.46	9.87	0.12	0.34	1.11	0.00
	VS041	44/VS95/0-1/36/13	2nd half 11th c.	greenish	undetermined body fragment	17.4	3.56	1.80	64.1	0.42	1.20	1.45	8.69	0.09	0.26	0.83	0.01
	VS043	46/VS96/W ALC/2	10th - 11th c.	purple tinge	small bottle neck fragment	18.6	4.27	1.47	63.1	0.71	1.33	1.99	6.51	0.07	0.60	0.56	0.63
	VS056	60/VS97/0-5/15B/30	2nd half 11th - 1st 1/4 12th c.		undetermined body fragment	16.2	3.81	1.61	64.8	0.45	1.11	1.78	8.55	0.08	0.68	0.73	0.07
	VS065	74/VS98/0-5/10/16 Bajo tejas y 54	11th - 12th c.	dark blue	bottle base	19.2	5.36	1.30	63.0	0.44	1.20	1.72	5.31	0.06	0.03	0.51	0.03
	VS078	89/VS98/0-5/17F/146	2nd half 11th - 1st 1/4 12th c.	purple tinge	bottle base	17.8	4.70	2.26	62.5	0.51	1.04	1.90	5.66	0.09	0.90	0.75	1.55
	VS093	107/VS001/0-5/B9/579	2nd half 11th - 1st 1/4 12th c.	amber	bowl base ?	19.5	4.95	1.77	59.6	0.91	1.03	2.73	7.50	0.09	0.89	0.77	0.00
	VS114	127/VS07/PORTILLO 2/INT/C-2/2	10th - 11th c.	light blue	bowl rim ?	18.4	3.61	2.06	60.8	0.46	0.94	1.61	4.51	0.08	0.06	0.88	4.72
	VS118	131/VS07/PUERTA S/Ext Amp/1	10th - 11th c.	colourless	jewellery	18.8	4.39	2.04	62.4	0.49	1.14	2.02	6.79	0.09	0.47	0.70	0.50
	VS120	133/VS07/PORTILLO 2/INT/C-3/2	10th - 11th c.	colourless	unguentaria base	17.4	6.18	2.08	64.5	0.58	1.12	2.18	5.06	0.07	0.06	0.43	0.00
Group 4	VS002	2/VS78/0-1/s.UE	Superficial		bottle base	20.3	4.85	6.01	55.8	0.69	1.08	2.29	6.70	0.30	0.61	1.10	0.05
	VS007	9/VS86/PORTILLO/0-6/SEC 2/ II(a)	10th - 11th c.	deep purple	tubular unguentaria base	20.4	5.07	7.22	55.1	0.57	0.93	2.66	5.27	0.36	0.94	1.35	0.00
	VS009	10/VS86/PORTILLO/G-1/III(ba)	10th - 11th c.	deep purple	tubular unguentaria base	20.5	4.97	7.22	55.1	0.55	0.94	2.54	5.26	0.36	1.00	1.36	0.00
	VS010	11/VS86/PORTILLO/L-5/II	10th - 11th c.	colourless	tubular unguentaria base	19.7	5.09	4.35	58.5	0.51	1.20	2.04	7.24	0.22	0.27	0.68	0.00
	VS011	12/VS86/PORTILLO/I-3/SEC 1/ II (A-B)	10th - 11th c.	purple	tubular unguentaria base	20.5	4.36	4.71	57.7	0.52	1.15	1.94	7.07	0.21	0.66	0.75	0.07
	VS024	26/VS92/0-1/9/UE 9	10th - 11th c.	purple tinge	unguentaria rim	19.7	3.81	4.59	60.5	0.42	1.23	2.03	5.94	0.23	0.58	0.78	0.00
	VS048	51/VS96/0-5/9/3	11th c.	colourless	unguentaria rim and body fragment	20.0	3.77	3.98	59.5	0.51	1.19	2.25	6.84	0.26	0.55	0.90	0.00
	VS055	59/VS07/0-5/O. Aljibe/2	11th c.	colourless	undetermined body fragment	19.4	5.74	5.65	55.3	1.15	0.97	3.04	6.67	0.29	0.48	1.11	0.00
	VS059	65/VS97/0-5/15B/30	11th c.	colourless	tubular unguentaria base	18.1	4.28	4.44	59.2	1.05	1.10	2.71	6.64	0.31	0.47	1.45	0.00
	VS064	73/VS98/0-5/10/118	2nd half 11th - 1st 1/4 12th c.	purple tinge	undetermined body fragment	19.4	4.28	5.78	57.1	0.68	1.09	2.24	6.65	0.28	0.70	1.07	0.49
	VS067	76/VS98/0-5/10/54	2nd half 11th - 1st 1/4 12th c.	light green	unguentaria base	18.6	5.35	5.23	56.9	0.67	1.05	2.37	8.08	0.25	0.14	0.88	0.34
	VS069	78/VS98/0-5/10/18 Sobre suelo	11th - 1st 1/4 12th c.	greenish	unguentaria rim	18.6	5.35	5.24	56.8	0.67	1.04	2.38	8.15	0.25	0.13	0.89	0.25
	VS071	66/VS98/0-5/15C/115	11th - 1st 1/4 12th c.	light green	unguentaria base and body	18.8	3.65	3.76	60.6	0.56	1.17	2.22	7.13	0.25	0.51	1.10	0.09
	VS077	88/VS98/0-5/16E/NS	Superficial	colourless	decorative cut flat glass	18.6	3.91	4.64	60.4	0.47	1.08	2.19	6.43	0.24	0.21	0.88	0.79
	VS082	93/VS98/0-5/13I/NS	Superficial	greenish	unguentaria rim	18.2	3.71	3.52	62.7	0.51	1.20	1.94	6.90	0.25	0.06	0.87	0.01
	VS089	100/VS98/0-5/14E/NS	Superficial	purple	unguentaria base	19.2	4.88	4.40	59.0	0.58	1.11	2.16	6.25	0.23	0.54	0.71	0.75
	VS098	112/VS001/0-5/F5/440	2nd half 11th - 1st 1/4 12th c.	deep purple	unguentaria base	19.8	4.24	5.23	58.5	0.44	1.21	1.90	6.53	0.25	0.59	0.82	0.31
	VS103	117/VS001/0-4/2F/34	10th - 11th c.	light green	possibly lamp base	17.2	4.35	4.83	60.4	0.71	0.70	2.64	6.86	0.32	0.69	1.07	0.01
	VS109	124/VS07/PUERTA S/Ext Amp/1	Superficial	greenish	unguentaria base	19.3	4.19	3.94	59.5	0.46	1.05	2.23	6.71	0.20	0.36	0.76	0.82
	VS117	130/VS07/PORTILLO 2/INT/C-3/1	Superficial	amber	unguentaria rim	18.5	5.55	5.21	56.3	1.09	1.10	2.69	8.24	0.27	0.08	0.85	0.00
	VS121	144/VS08/PORTILLO III/EXT/UE 6	10th - 11th c.	colourless	unguentaria body fragment	19.4	4.09	6.06	59.0	0.51	1.10	2.19	5.08	0.32	0.82	1.18	0.09
	VS122	145/VS08/PUERTA S/20	10th - 11th c.	colourless	unguentaria handle, applied decoration	19.5	3.28	4.29	60.6	0.64	1.17	2.49	5.92	0.30	0.66	0.97	0.03
	VS125	148/VS08/PUERTA W/2	10th - 11th c.	purple	possibly lamp base	19.6	4.08	5.11	58.4	0.61	1.17	2.10	7.14	0.32	0.37	0.87	0.00
	VS131	68/VS98/0-5/10/29	2nd half 11th - 1st 1/4 12th c.	purple tinge	undetermined body fragment	19.2	4.25	5.66	57.3	0.69	1.11	2.27	6.73	0.29	0.70	1.08	0.48
	VS132	150/VS6/BASURERO S/1/CAPA 2/Nº12	2nd half 11th c.	light green	unguentaria base	20.2	3.75	4.19	60.8	0.44	1.24	2.22	4.91	0.29	0.38	1.08	0.32
	VS134	152/VS03/0-4/R13/106/nº5	2nd half 11th - 1st 1/4 12th c.	greenish	small bottle base	17.9	5.07	4.38	58.7	0.98	1.13	2.83	7.06	0.26	0.52	0.98	0.00
	VS135	153/VS05/0-4/Calle/92B/NS/nº4	Superficial	greenish	unguentaria base	20.0	3.96	6.10	57.3	0.52	1.18	2.29	6.65	0.32	0.26	1.32	0.00
	VS136	154/VS06/BASURERO S/2/CAPA 2/nº 2	2nd half 11th c.	colourless	small bottle rim	19.3	4.86	4.59	58.9	0.51	1.17	1.85	7.07	0.22	0.45	0.73	0.16
	VS137	155/VS06/BASURERO S/6/CAPA 2	2nd half 11th c.	light green	undetermined base	16.2	3.66	4.79	61.8	0.54	0.91	2.57	7.04	0.33	0.85	1.11	0.01
	VS140	158/VS03/0-4/Patio/181/nº18	11th c.	bluish	undetermined body fragment	16.1	4.87	5.10	60.7	0.61	0.92	2.50	5.86	0.21	0.72	1.11	0.26
	VS142	160/VS06/0-4/Calle Sur/91B/521, nº22	11th c.	greenish	undetermined body fragment	17.9	5.23	4.42	58.5	0.97	1.09	2.83	7.25	0.26	0.46	0.97	0.00

Sample	ppm																																												
Sample	Li	B	V	Cr	Co	Ni	Cu	Zn	Ga	As	Rb	Sr	Y	Zr	Nb	Mo	Ag	Cd	In	Sn	Sb	Cs	Ba	La	Ce	Pr	Nd	Sm	Eu	Gd	Tb	Dy	Ho	Er	Tm	Yb	Lu	Hf	Ta	W	Pt	Au	Bi	Th	U
VS028	6.09	88.4	8.79	12.1	2.11	6.67	24.3	30.0	2.91	1.69	14.9	546	5.42	32.2	1.23	2.42	0.07	0.04	0.00	0.27	0.10	0.13	230	5.52	9.31	1.19	4.86	0.97	0.29	0.91	0.16	0.82	0.17	0.50	0.06	0.48	0.07	0.83	0.07	0.26	0.00	0.00	0.01	0.71	0.37
VS066	4.88	80.7	12.3	12.4	3.94	8.61	11.1	27.1	3.64	2.67	14.9	437	5.99	38.1	1.81	1.93	0.06	0.07	0.00	0.36	0.01	0.14	239	5.95	10.6	1.32	5.65	1.13	0.31	1.13	0.18	0.97	0.20	0.54	0.08	0.49	0.08	0.95	0.10	0.12		0.00	0.01	0.86	0.39
VS076	5.48	66.7	13.4	17.3	3.70	9.18	16.2	30.5	3.75	1.88	12.9	627	6.56	42.2	2.00	1.92	0.10	0.06	0.00	1.19		0.11	273	6.86	12.1	1.46	5.87	1.17	0.33	1.10	0.19	1.00	0.22	0.64	0.08	0.54	0.08	1.08	0.11				0.01	0.99	0.56
VS085	5.46	62.9	13.6	16.5	3.76	9.24	15.6	34.5	3.79	2.59	12.2	658	6.96	44.4	1.99	1.76	0.10	0.07	0.01	1.15	0.14	0.11	284	7.03	12.4	1.51	6.38	1.24	0.34	1.19	0.20	1.08	0.23	0.63	0.08	0.59	0.09	1.15	0.12	0.21		0.00	0.01	1.00	0.56
VS090	5.62	61.9	13.7	16.0	3.82	9.07	15.8	35.7	3.75	2.74	12.0	655	6.78	43.8	2.00	1.70	0.11	0.05	0.00	1.30	0.24	0.11	293	7.02	12.3	1.54	6.27	1.30	0.35	1.21	0.20	1.09	0.22	0.63	0.09	0.59	0.09	1.08	0.12	0.20		0.01	0.01	1.05	0.56
VS105	4.72	72.9	11.3		3.11	7.46	34.1	21.7	5.25	18.6	28.2	559	5.37	33.5	1.46	2.69	1.09	0.04	0.00	0.46		0.11	209	5.18	9.32	1.23	5.31	1.11	0.34	1.30	0.19	0.94	0.19	0.54	0.07	0.46	0.07	0.83	0.08	0.31	0.00		0.01	0.69	0.37
VS111	4.94	78.0	12.6		3.41	8.28	34.1	26.4	3.67		12.9	448	6.00	35.5	1.52	2.44	0.09	0.05	0.00	0.20		0.10	220	5.86	11.2	1.42	6.11	1.30	0.37	1.38	0.20	1.03	0.21	0.59	0.07	0.51	0.07	0.88	0.09	0.29	0.00	0.18		0.76	0.38
VS130	5.06	80.7	10.9	4.20	3.93	6.77	25.2	21.5	3.18	2.51	14.3	452	6.02	33.4	1.47	2.62	0.10	0.03	0.00	0.46		0.13	223	6.10	10.7	1.32	5.50	1.12	0.31	1.00	0.17	0.88	0.20	0.55	0.07	0.47	0.07	0.83	0.08	0.14	0.00	0.00	0.01	0.79	0.34
VS144	5.92	71.9	12.8		3.36	7.41	37.6	27.5	3.77	2.39	14.5	509	7.33	38.5	1.58	2.70	0.06	0.04	0.01	0.37		0.13	255	7.95	14.7	1.78	7.56	1.44	0.40	1.12	0.23	1.16	0.24	0.66	0.08	0.58	0.09	0.95	0.10	0.29	0.00	0.00	0.01	0.95	0.41
VS147	5.08	64.5	12.4		3.70	7.94	12.0	29.8	3.84	2.01	12.7	436	6.71	39.7	1.77	2.31	0.05	0.05	0.00	0.50		0.12	276	7.46	13.5	1.57	6.28	1.22	0.34	0.95	0.18	1.08	0.22	0.62	0.08	0.52	0.08	1.03	0.10	0.19	0.00	0.00	0.00	0.99	0.41
VS016	56.1	134	8.69	11.5	1.69	7.35	28.3	49.6	2.77	2.05	7.19	1008	3.77	31.7	1.60	0.21	0.38	0.06	0.00	151	0.78	0.20	59.9	5.34	10.1	1.12	4.38	0.84	0.19	0.91	0.13	0.67	0.14	0.41	0.05	0.38	0.06	0.86	0.12	0.03	0.00		0.04	1.59	0.65
VS026	40.6	133	13.8	12.4	3.35	9.19	226	67.5	5.17	4.54	13.1	886	5.75	50.2	2.21	3.82	0.84	0.05	0.01	15.7	3.90	0.37	237	7.59	14.4	1.66	6.22	1.19	0.28	1.18	0.19	0.99	0.20	0.58	0.08	0.55	0.08	1.29	0.15	0.12			0.17	2.16	1.61
VS039	50.1	136	14.4	15.5	3.22	9.05	24.1	54.6	4.49	2.35	8.01	1031	6.28	49.4	2.28	0.35	0.11	0.06	0.01	1.02		0.25	98.2	7.98	15.3	1.71	6.78	1.41	0.29	1.45	0.22	1.05	0.23	0.66	0.09	0.60	0.09	1.28	0.16	0.07	0.00	0.00	0.06	2.21	1.14
VS041	42.3	161	9.10	12.0	2.29	10.8	26.0	61.8	3.36	1.02	9.04	729	4.64	39.3	1.72	0.61	0.12	0.04	0.01	1.02		0.19	75.8	5.69	11.0	1.25	4.90	0.99	0.21	1.04	0.16	0.83	0.17	0.48	0.07	0.46	0.07	1.01	0.13	0.06	0.00	0.00	0.02	1.63	0.68
VS043	42.4	176	6.42	8.63	2.20	26.3	337	93.1	3.04	2.17	7.71	295	3.40	27.3	1.37	0.43	0.56	0.03	0.02	17.3	5.39	0.14	99.4	4.54	8.45	1.00	3.91	0.82	0.15	0.83	0.12	0.59	0.12	0.36	0.05	0.34	0.05	0.69	0.10	0.04	0.00	0.00	0.32	1.40	0.62
VS056	30.3	161	8.43	9.92	1.93	8.29	31.4	54.3	3.07	2.29	6.88	703	4.69	34.6	1.56	1.80	0.10	0.06	0.00	7.34	1.31	0.16	121	5.61	10.7	1.23	4.80	1.00	0.21	0.96	0.15	0.82	0.16	0.48	0.07	0.46	0.06	0.92	0.12	0.04	0.00	0.00	0.06	1.54	0.81
VS065	51.4	216	4.59	7.08	2.43	23.1	12770	159	2.18	10.8	5.37	294	2.58	15.2	1.10	0.16	3.46	0.07	0.01	36.8	22.7	0.11	35.8	3.49	6.56	0.76	2.98	0.58	0.12	0.57	0.09	0.46	0.09	0.27	0.04	0.25	0.04	0.40	0.09	0.02	0.00	0.07	1.72	1.08	0.39
VS078	53.3	187	7.54	9.17	3.20	19.6	498	156	4.22	5.90	14.1	324	4.75	38.8	1.71	1.87	0.87	0.07	0.14	5.93	51.9	0.28	513	7.10	13.4	1.55	6.13	1.19	0.24	1.02	0.16	0.82	0.16	0.45	0.07	0.44	0.07	1.05	0.14				0.66	2.48	1.13
VS093	48.9	190	9.58	9.79	4.89	23.4	131	99.3	3.39	2.64	9.38	498	4.37	33.8	1.71	1.83	0.30	0.03	0.01	1.91	0.26	0.16	103	6.08	11.3	1.28	4.90	0.98	0.21	0.91	0.14	0.76	0.15	0.42	0.06	0.43	0.06	0.90	0.11	0.17	0.00	0.00	0.03	1.66	0.90
VS114	47.9	160	8.17		4.80	20.2	13031	123	3.25	12.1	11.1	242	4.15	30.1	1.43	0.34	5.04	0.05	0.71	240	124	0.24	639	6.13	11.5	1.38	5.48	1.13	0.30	1.04	0.16	0.76	0.15	0.42	0.06	0.39	0.06	0.79	0.10	0.44	0.00	0.09	4.48	1.89	0.93
VS118	44.0	163	8.01	2.72	2.66	18.0	289	107	3.46	5.58	11.7	341	4.40	34.9	1.68	0.64	1.02	0.06	0.07	5.65	21.8	0.22	307	6.48	12.5	1.46	5.70	1.12	0.26	0.96	0.15	0.76	0.15	0.43	0.06	0.41	0.06	0.93	0.12	0.06	0.00	0.00	0.22	1.94	0.74
VS120	45.5	221	4.14		2.04	26.2	912	77.8	3.15	1.81	14.1	407	4.17	40.1	1.45	0.31	0.22	0.04	0.00	19.5	1.09	0.36	64.6	6.95	13.0	1.50	5.75	1.08	0.20	0.96	0.14	0.70	0.15	0.43	0.06	0.40	0.06	1.06	0.15	0.06	0.00	0.00	0.53	2.34	0.68
VS002	50.9	224	20.7	19.7	3.41	32.4	34.7	80.7	8.36		11.1	434	9.96	152	5.37	1.53	0.47	0.03	0.01	1.88	0.26	0.32	147	14.5	27.8	3.29	12.8	2.51	0.51	2.86	0.38	1.77	0.37	1.11	0.15	1.14	0.17	3.85	0.38	0.09	0.01	0.00	0.07	4.62	1.85
VS007	99.1	230	26.4	21.5	14.7	29.8	59.2	65.8	10.4	10.6	11.6	312	13.5	168	6.30	2.26	0.47	0.05	0.02	2.05		0.36	164	18.1	36.9	4.07	16.3	3.35	0.65	3.69	0.51	2.40	0.50	1.49	0.20	1.46	0.21	4.24	0.47	0.11	0.01		0.31	5.80	2.65
VS009	96.4	228	26.8	22.4	14.9	29.2	55.3	65.1	10.5	10.6	11.9	313	13.7	169	6.33	2.51	0.47	0.07	0.02	2.16		0.35	174	18.2	37.1	4.11	16.3	3.26	0.65	3.69	0.52	2.39	0.49	1.49	0.19	1.47	0.21	4.25	0.51	0.13	0.01		0.31	5.84	2.71
VS010	52.9	231	15.0	15.2	2.22	23.0	29.4	64.6	5.92		7.30	380	8.11	128	3.96	0.74	0.37	0.04	0.01	1.01		0.19	86.7	11.3	21.5	2.49	10.0	1.83	0.38	2.20	0.30	1.37	0.30	0.91	0.12	0.90	0.13	3.18	0.29	0.09	0.01		0.01	3.54	1.41
VS011	45.9	178	15.0	14.0	2.86	19.2	1548	59.0	6.10	0.26	7.50	273	6.50	125	3.83	1.21	1.49	0.04	0.01	3.30	4.14	0.19	181	9.65	18.4	2.12	8.46	1.57	0.33	1.74	0.23	1.12	0.23	0.76	0.10	0.79	0.12	3.06	0.28	0.10	0.01	0.02	0.13	3.46	1.44
VS024	47.2	193	16.6	17.2	2.37	18.7	355	59.6	6.53	2.55	9.57	261	8.40	113	4.13	1.58	0.33	0.02	0.01	5.70	0.80	0.25	131	12.1	23.1	2.59	10.1	1.91	0.39	1.92	0.28	1.50	0.30	0.91	0.12	0.89	0.14	2.86	0.30	0.10	0.00		0.08	3.82	1.66
VS048	48.1	187	16.7	18.9	8.30	22.9	277	61.9	6.65	8.74	12.8	329	10.1	152	4.46	1.18	0.40	0.05	0.01	1.31	0.29	0.36	108	13.5	27.7	3.07	12.2	2.43	0.50	2.48	0.35	1.79	0.37	1.04	0.15	1.07	0.15	3.75	0.32	0.15	0.01	0.00	0.21	4.00	1.99
VS055	46.2	180	21.0	21.3	8.13	35.3	72.0	108	8.81	8.89	12.8	354	11.2	138	5.16	4.25	0.29	0.06	0.01	2.12	0.59	0.47	149	15.1	30.4	3.42	13.2	2.65	0.53	2.68	0.39	2.00	0.41	1.13	0.17	1.14	0.17	3.53	0.38	0.15	0.00	0.00	0.25	4.76	1.92
VS059	24.7	168	18.5	22.1	6.73	15.9	119	121	7.23	3.31	15.7	328	9.62	179	4.89	0.37	0.41	0.11	0.02	1.91	2.73	0.44	144	13.5	26.0	2.89	11.4	2.15	0.43	2.17	0.31	1.65	0.36	1.01	0.14	1.09	0.15	4.31	0.33	0.47	0.01	0.00	0.07	4.00	1.07
VS064	49.4	200	21.6	22.4	3.72	27.4	46.3	71.7	8.48	14.1	13.7	315	9.52	133	5.12	2.05	1.30	0.04	0.02	29.5	20.5	0.41	171	14.4	28.0	3.16	12.3	2.29	0.46	2.24	0.31	1.58	0.35	1.00	0.14	1.05	0.16	3.39	0.35	0.14	0.01	0.00	0.25	4.52	2.01
VS067	57.8	197	17.6	20.9	2.80	16.9	292	58.7	7.14	21.0	10.3	415	8.42	134	4.53	0.38	1.34	0.03	0.02	17.2	18.9	0.28	110	13.2	25.5	2.87	11.0	2.06	0.40	1.78	0.27	1.50	0.31	0.90	0.13	0.94	0.15	3.34	0.32				0.17	4.30	1.51
VS069	57.6	195	17.7	21.1	2.78	16.9	300	58.5	7.04	15.7	9.88	422	8.30	134	4.57	0.44	1.32	0.05	0.02	12.4	14.4	0.26	109	13.0	25.3	2.80	10.8	1.97	0.40	1.78	0.28	1.46	0.31	0.89	0.13	0.93	0.14	3.39	0.33				0.13	4.26	1.51
VS071	38.4	153	16.5	17.4	4.88	19.0	59.4	64.7	6.39	5.78	11.3	284	9.29	143	4.38	1.58	0.29	0.03	0.01	6.49	1.52	0.29	107	13.7	27.3	2.97	11.7	2.25	0.45	1.98	0.30	1.64	0.33	0.96	0.14	0.99	0.15	3.59	0.32				0.14	4.19	1.94
VS077	50.9	177	17.1	17.2	3.18	15.5	158	66.9	6.98	28.5	16.6	287	8.84	171	4.37	0.64	2.14	0.03	0.05	38.8	29.1	0.37	123	13.6	26.1	2.96	11.3	2.04	0.41	1.89	0.29	1.52	0.32	0.92	0.14	0.97	0.15	4.27	0.31				0.48	4.37	1.94
VS082	38.5	155	13.8	16.4	4.87	17.6	24.7	56.5	5.77	4.93	8.99	289	9.03	139	4.34	0.26	0.19	0.05	0.01	1.23	0.63	0.23	61.5	13.0	26.4	2.90	11.2	2.19	0.47	1.90	0.30	1.56	0.32	0.92	0.14	0.98	0.15	3.55	0.32	0.14	0.00	0.00	0.12	4.16	2.10
VS089	61.3	181	14.1	16.1	2.74	15.5	87.3	70.8	6.51	13.0	10.5	351	8.14	124	4.10	1.37	1.52	0.06	0.00	34.1	29.7	0.24	159	12.0	23.0	2.59	9.91	1.89	0.36	1.72	0.26	1.37	0.30	0.87	0.12	0.86	0.14	3.16	0.30	0.10	0.00		0.83	3.79	1.49
VS098	57.8	211	18.1	17.9	2.66	19.1	29.2	61.3	7.61	7.39	11.1	305	8.87	125	4.54	1.59	0.76	0.06	0.02	16.4	9.06	0.25	137	13.6	26.1	2.90	11.3	2.06	0.40	1.84	0.29	1.58	0.32	0.92	0.14	0.95	0.15	3.15	0.32	0.08	0.00		0.12	4.30	1.94
VS103	45.1	193	20.6	20.2	6.32	21.9	23.9	84.0	8.60	5.17	13.6	383	13.2	196	5.63	2.03	0.28	0.06	0.01	1.77	0.71	0.39	146	17.9	36.4	3.97	15.1	3.05	0.61	2.54	0.42	2.30	0.49	1.38	0.20	1.36	0.20	4.91	0.41	0.25	0.00	0.00	0.07	5.66	2.23
VS109	49.2	182	14.2		2.23	15.3	2390	57.8	5.55	38.3	12.0	313	7.72	124	3.54	0.91	5.33	0.04	0.02	35.8	31.1	0.25	127	10.7	20.7	2.36	9.21	1.76	0.36	1.89	0.27	1.34	0.28	0.84	0.12	0.83	0.13	3.14	0.26	0.12	0.00	0.03	0.63	3.45	1.37
VS117	45.0	195	13.0	17.9	2.77	19.7	118	62.4	7.14	1.18	11.4	429	9.43	139	4.36	0.31	0.38	0.04	0.01	1.21		0.32	94.2	14.2	27.3	3.04	11.6	2.13	0.43	2.04	0.31	1.62	0.33	0.97	0.14	0.98	0.15	3.48	0.34	0.09	0.00	0.00	0.03	4.34	1.54
VS121	61.9	184	27.6		13.6	26.0	56.8	52.4	9.73	19.0	13.7	281	12.7	148	5.74	2.20	0.33	0.05	0.03	2.10		0.46	159	17.8	36.3	3.92	15.0	3.01	0.60	2.63	0.42	2.20	0.46	1.30	0.18	1.23	0.19	3.77	0.43	0.16	0.00		0.29	5.48	2.55
VS122	35.5	166	18.4		8.71	22.3	47.5	62.7	7.54	11.3	12.9	241	11.2	159	5.15	1.48	0.28	0.06	0.01	1.45		0.40	95.4	15.8	32.6	3.52	13.6	2.75	0.58	2.26	0.38	1.92	0.39	1.13	0.16	1.13	0.17	4.00	0.39	0.20	0.00	0.00	0.17	4.78	2.36
VS125	58.9	213	18.2	5.63	2.60	29.0	36.3	58.8	7.69	2.99	13.0	279	11.6	233	5.55	0.98	0.37	0.05	0.01	1.32		0.37	214	16.8	32.8	3.62	13.9	2.67	0.54	2.29	0.38	2.01	0.42	1.21	0.18	1.27	0.19	5.75	0.41	0.13	0.01	0.00	0.02	5.27	1.99
VS131	47.7	191	22.1	10.5	3.72	27.1	45.9	72.6	8.73	14.1	13.8	318	9.80	139	5.19	2.00	1.30	0.04	0.04	29.2	20.3	0.40	171	15.5	29.7	3.34	12.6	2.32	0.47	2.04	0.32	1.72	0.35	1.04	0.15	1.03	0.16	3.43	0.36	0.12	0.00	0.01	0.25	4.94	2.10
VS132	47.7	196	17.2	4.81	9.37	18.6	343	83.7	7.04	15.9	12.3	259	10.9	142	5.02	0.83	0.63	0.06	0.12	2.18	9.02	0.36	98.7	16.2	32.9	3.55	13.7	2.65	0.55	2.32	0.37	1.94	0.39	1.11	0.16	1.15	0.18	3.63	0.38	0.22	0.00	0.00	0.35	4.96	2.75
VS134	34.6	166	17.2		5.61	27.0	42.9	93.0	7.14	6.99	10.2	365	9.85	137	4.60	1.33	0.23	0.02	0.01	1.60	0.22	0.38	122	14.2	28.6	3.15	12.0	2.31	0.46	1.98	0.33	1.73	0.36	1.03	0.14	1.03	0.15	3.46	0.34	0.12	0.00	0.00	0.11	4.35	1.61
VS135	55.0	179	27.8	6.29	12.3	26.5	52.4	57.4	9.31	14.9	11.7	286	12.8	139	5.80	0.47	0.28	0.03	0.02	2.03	0.41	0.43	115	18.2	36.8	3.96	15.2	2.97	0.61	2.56	0.42	2.21	0.46	1.31	0.19	1.28	0.19	3.55	0.44	0.09	0.01	0.00	0.34	5.73	2.26
VS136	62.1	193	14.9	6.38	2.40	18.8	25.8	56.7	6.12	7.55	8.81	388	7.59	117	4.01	1.01	0.92	0.03	0.01	7.34	6.39	0.22	118	11.5	21.8	2.44	9.32	1.75	0.37	1.52	0.25	1.32	0.27	0.80	0.11	0.84	0.12	2.93	0.29	0.07	0.00	0.01	0.09	3.88	1.70
VS137	46.6	192	21.9	9.70	6.34	18.7	26.2	63.3	8.41	7.08	14.3	386	13.3	204	5.81	2.28	0.38	0.04	0.01	1.94	0.50	0.47	148	18.5	37.3	3.98	15.3	3.02	0.60	2.60	0.43	2.29	0.48	1.37	0.19	1.38	0.20	5.11	0.42	0.24	0.00	0.00	0.12	5.83	2.29
VS140	57.1	211	12.9		3.84	19.9	6586	99.6	7.45	25.4	28.7	328	8.40	99.7	3.57	2.02	7.25	0.10	0.06	177	27.0	0.70	281	13.8	27.0	3.04	11.6	2.20	0.40	1.77	0.30	1.49	0.31	0.84	0.11	0.81	0.13	2.52	0.31	0.22	0.00	0.15	2.12	5.44	1.56
VS142	37.3	164	17.1		5.61	27.0	44.7	95.0	7.09	6.74	11.7	371	10.1	149	4.57	1.19	0.22	0.02	0.01	1.58	0.25	0.37	124	15.1	30.0	3.24	12.5	2.48	0.49	1.93	0.33	1.78	0.36	1.07	0.15	1.04	0.16	3.85	0.35	0.12	0.01	0.00	0.12	4.66	1.69

	Sample	REF	Context date	Colour	Type	wt%											
	Sample	REF	Context date	Colour	Type	Na_2O	MgO	Al_2O_3	SiO_2	P_2O_5	Cl	K_2O	CaO	TiO_2	MnO	Fe_2O_3	PbO
	VS003	3/VS81/C-5/entre tejas	11th c.	purple tinge	bottle body fragment ?	17.1	5.63	2.24	61.9	0.44	0.95	1.97	4.59	0.10	1.26	0.67	2.84
	VS004	4/VS81/0-1/entre tejas (baños?)	11th c.	light blue	undetermined body fragment	18.5	3.94	3.08	61.1	0.67	1.06	2.40	6.05	0.11	0.54	0.78	0.53
	VS013	15/VS87/0-1/UE 156	10th - 11th c.	purple tinge	tubular unguentaria base	18.0	5.58	3.91	61.2	0.53	0.98	2.65	5.37	0.15	0.68	0.68	0.08
	VS025	27/VS92/0-1/6/UE 9	10th - 11th c.	purple tinge	small bottle base	18.1	4.06	3.06	63.3	0.72	1.00	2.57	5.31	0.16	0.75	0.71	0.00
	VS029	31/VS93/0-1/15/UE 4	2nd half 11th - 1st 1/4 12th c.	bluish	bottle / bowl body fragment	19.9	3.54	2.61	61.5	0.56	1.22	2.32	6.54	0.15	0.31	0.72	0.25
	VS037	40/VS95/0-1/38/3	2nd half 11th c.	dark blue	bottle / bowl body fragment	15.0	5.37	2.23	60.1	0.33	1.05	1.26	3.73	0.10	0.04	0.87	7.15
	VS044	47/VS96/0-1/BARBACANA/12	10th - 11th c.	bluish	undetermined body fragment	16.9	4.00	3.55	58.0	0.50	1.09	2.17	6.88	0.14	0.16	1.26	3.58
	VS049	52/VS96/0-5/O Barbacana/2	2nd half 11th - 1st 1/4 12th c.	colourless	bottle rim	15.3	4.27	3.54	60.8	0.68	0.87	2.52	8.31	0.16	0.99	0.85	1.49
	VS052	56/VS96/0-5/2/6	Before mid 11th c.	colourless	decorative cut flat glass	16.9	3.65	5.46	60.0	0.54	1.10	1.90	8.68	0.17	0.49	0.80	0.21
	VS053	57/VS97/0-1/40/22	10th - 11th c.	turquoise	unguentaria (olliforme) rim	14.9	5.32	2.22	60.3	0.33	1.10	1.23	3.69	0.10	0.03	0.86	7.19
	VS058	62/VS97/0-5/C-5/5	2nd half 11th c.	purple tinge	undetermined body fragment	17.7	3.77	3.99	61.3	0.48	1.14	2.33	7.13	0.14	0.86	0.76	0.20
	VS061	67/VS98/0-5/10/118	2nd half 11th - 1st 1/4 12th c.	colourless	unguentaria base	17.9	4.77	4.53	60.1	0.55	1.09	2.51	5.72	0.16	0.43	0.89	0.96
	VS063	72/VS98/0-5/10/5	2nd half 11th - 1st 1/4 12th c.	dark green	jewellery	14.4	4.34	3.85	56.7	0.45	0.70	1.90	4.99	0.15	0.23	1.25	8.17
	VS073	84/VS98/0-5/A/3/4	2nd half 11th c.	colourless	bowl base ?	17.7	5.04	2.89	60.9	0.57	1.06	1.82	6.94	0.17	1.04	1.44	0.00
Group 5	VS079	90/VS98/0-5A/17D/146	2nd half 11th - 1st 1/4 12th c.	colourless	undetermined body fragment	12.2	4.39	3.31	67.5	0.81	1.32	2.59	6.43	0.13	0.45	0.65	0.00
	VS080	91/VS98/0-5A/15G/NS	10th - 11th c.	colourless	small bottle base	18.3	4.00	1.93	64.5	0.49	1.19	2.00	4.80	0.10	0.31	0.60	1.58
	VS081	92/VS98/0-5A/14G/NS	Superficial	colourless	unguentaria base	14.1	3.66	2.81	65.6	0.44	0.84	2.06	7.11	0.13	0.42	0.97	1.52
	VS094	108/VS001/0-5/E2/880	2nd half 11th - 1st 1/4 12th c.	purple	bottle rim	17.6	3.73	3.90	61.7	0.49	1.10	2.29	7.06	0.14	0.86	0.76	0.17
	VS102	116/VS001/0-4/2E/5	11th c.	greenish	unguentaria base	18.6	4.47	4.36	60.3	0.59	1.18	2.01	7.38	0.17	0.20	0.55	0.02
	VS110	125/VS07/PUERTA S/Ext Amp/1	Superficial	purple tinge	bottle base	20.4	3.59	3.67	61.0	0.48	1.34	2.00	5.80	0.14	0.83	0.54	0.06
	VS112	135/VS07/PUERTA S/Ext Amp/NS	Superficial	colourless	bottle base	18.2	5.12	2.91	62.8	0.51	1.13	2.19	5.57	0.12	0.30	0.69	0.04
	VS113	126/VS07/PORTILLO 2/INT/C-2/2	10th - 11th c.	yellowish	undetermined body fragment	18.5	4.01	2.65	60.6	0.44	1.12	1.98	9.00	0.24	0.11	0.66	0.43
	VS115	128/VS07/PORTILLO 1/INT/12	11th c.	colourless	undetermined body fragment	13.2	2.67	2.28	67.0	0.40	0.75	2.23	9.23	0.14	0.86	0.87	0.14
	VS116	129/VS07/PORTILLO 2/INT/C-3/1	Superficial	bluish	unguentaria rim	18.1	4.45	2.96	57.0	0.71	1.07	2.36	8.13	0.15	0.12	0.81	0.11
	VS119	132/VS07/PORTILLO 2/INT/C-3/6	10th - 11th c.	colourless	bottle base	18.3	4.40	3.53	56.8	0.57	0.89	2.39	6.55	0.14	0.87	0.80	4.51
	VS133	151/VS05/0-4/Sur Este/99H/NS/nº3	Superficial	dark blue	bottle rim	14.5	5.21	2.13	60.8	0.33	1.11	1.22	3.66	0.10	0.04	0.85	7.31
	VS138	156/VS06/Sin contexto	Superficial	dark blue	bottle base	15.7	5.15	2.17	60.5	0.57	0.98	2.04	5.28	0.11	0.28	0.71	4.47
	VS141	159/VS06/0-4/Calle Sur/91C/R17/493/nº17	2nd half 11th c.	greenish	undetermined body fragment	11.5	2.69	2.66	66.0	0.54	0.78	2.20	10.9	0.18	0.99	1.10	0.23
Soda ash lead glass																	
	VS032	34/VS93/0-1/13/UE NS	Superficial	greenish	undetermined body fragment	5.18	0.84	0.74	39.2	0.26	2.26	0.48	1.26	0.04	0.43	0.46	48.5
	VS054	58/VS9770-5A/1	Superficial	yellowish	bottle rim ?	6.58	1.47	1.31	42.8	0.50	1.79	0.82	2.46	0.06	0.93	0.63	40.1
	VS108	123/VS05/0-4/Calle/95C/1/nº2	10th - 11th c.	dark green	small bottle rim	5.95	1.05	1.38	39.3	0.50	1.75	0.69	2.78	0.06	0.19	0.69	43.7
	VS123	146/VS08/PUERTA W/2	10th - 11th c.	colourless	possibly lamp base	5.90	1.32	1.44	38.7	0.32	2.12	0.41	1.96	0.07	0.64	0.66	45.9
	VS124	147/VS08/PUERTA W/2	10th - 11th c.	colourless	possibly lamp base	4.73	0.98	1.49	40.1	0.40	1.69	1.01	2.11	0.07	0.77	0.75	45.3
	VS139	157/VS06/0-4/Calle Sur/91C/R17/493/nº20	2nd half 11th c.	bluish	undetermined body fragment	4.78	1.02	0.58	40.5	0.25	2.46	0.55	1.21	0.03	0.03	0.32	47.3
Outliers																	
	VS012	14/VS86-87/0-1/S. UE	Superficial	bluish	bottle rim	16.1	4.34	3.11	63.2	0.58	0.93	3.69	3.55	0.11	0.65	1.03	0.06
	VS021	23/VS90/0-1/A-1/3/UE 16	10th - 11th c.	yellowish	undetermined mould blown body fragment	18.7	4.68	1.94	63.4	0.38	1.22	1.48	5.12	0.09	1.46	1.06	0.00
	VS027	29/ VS93/0-1/15/UE 1	10th - 11th c.	colourless	undetermined body fragment	16.8	3.41	1.29	59.9	0.97	0.84	5.41	8.71	0.05	1.32	0.85	0.01
	VS091	102/VS99/0-5/19H/239	10th - 11th c.	colourless	unguentaria body fragment	18.2	3.62	1.94	62.8	0.35	1.38	0.84	8.83	0.11	0.86	0.85	0.00
	VS092	106/VS01/0-5/H8/559	2nd half 11th c.	greenish	unguentaria base	16.8	5.02	7.23	53.4	1.17	0.76	6.10	4.70	0.26	0.10	2.36	1.80

	ppm																																														
Sample	Li	B	V	Cr	Co	Ni	Cu	Zn	Ga	As	Rb	Sr	Y	Zr	Nb	Mo	Ag	Cd	In	Sn	Sb	Cs	Ba	La	Ce	Pr	Nd	Sm	Eu	Gd	Tb	Dy	Ho	Er	Tm	Yb	Lu	Hf	Ta	W	Pt	Au	Bi	Th	U
VS003	107	221	7.29	6.56	2.72	12.7	545	68.3	4.63		10.2	310	5.27	69.6	1.89	0.83	1.22	0.09	0.09	6.11	50.7	0.20	394	8.06	15.7	1.89	7.67	1.53	0.32	1.72	0.23	0.95	0.20	0.58	0.08	0.53	0.08	1.82	0.16	0.18	0.00	0.00	0.46	3.11	2.56
VS004	40.0	161	9.17	10.3	2.71	15.7	8494	86.3	4.46	20.4	16.4	278	5.71	61.2	2.22	1.31	5.77	0.09	0.08	177	64.6	0.45	259	7.89	14.6	1.73	6.89	1.28	0.25	1.54	0.21	0.99	0.20	0.62	0.08	0.61	0.08	1.63	0.20	0.10	0.00	0.03	2.63	2.96	0.95
VS013	70.1	240	7.54	9.11	3.40	19.5	172	73.7	5.15		35.2	327	5.00	60.2	2.42	1.69	3.23	0.05	0.01	16.1	1.30	0.74	200	7.78	15.3	1.76	6.98	1.36	0.30	1.45	0.19	0.89	0.18	0.54	0.07	0.50	0.08	1.58	0.21	0.10	0.00	0.03	0.16	2.72	1.05
VS025	38.2	158	11.9	12.0	4.94	28.5	670	90.3	5.11	4.46	10.3	250	7.16	87.4	2.87	1.80	0.57	0.05	0.00	14.5	1.34	0.31	138	9.95	19.5	2.19	8.48	1.69	0.38	1.63	0.25	1.22	0.25	0.73	0.10	0.71	0.10	2.19	0.22	0.10	0.00		0.23	2.91	1.48
VS029	40.6	146	10.8	13.1	3.74	17.4	1844	74.6	4.40	9.88	13.5	281	6.39	83.3	2.66	0.78	2.22	0.06	0.02	87.6	16.0	0.35	89.7	9.07	17.8	1.97	7.62	1.47	0.29	1.31	0.21	1.09	0.22	0.64	0.09	0.65	0.10	2.15	0.21	0.27	0.00	0.03	0.72	2.92	1.10
VS037	83.6	191	8.88	8.18	3.99	17.4	19506	96.9	4.11	29.5	7.87	255	5.61	74.0	1.84	0.27	3.97	0.14	0.35	465	188	0.20	610	8.87	17.0	2.06	8.36	1.72	0.36	1.70	0.23	1.05	0.20	0.59	0.08	0.60	0.08	1.95	0.15	0.41	0.00	0.13	8.08	3.30	2.28
VS044	38.2	157	9.86	11.3	4.80	28.7	10459	571	5.83	39.9	21.5	406	6.44	79.8	2.58	0.28	4.30	0.34	0.55	394	218	0.40	1378	11.0	23.2	2.73	11.2	2.28	0.57	2.13	0.26	1.23	0.24	0.68	0.09	0.63	0.09	2.04	0.20	0.08	0.00	0.13	3.09	3.92	1.11
VS049	46.6	172	12.8	15.4	4.09	13.3	130	87.0	5.77	57.6	17.1	664	12.0	77.0	2.88	2.77	3.76	0.06	0.00	143	105	0.48	208	12.1	20.9	2.60	10.4	2.10	0.49	2.27	0.32	1.67	0.35	1.01	0.13	0.91	0.12	1.99	0.23	0.13	0.00	0.01	0.68	3.10	1.50
VS052	39.7	180	11.7	14.9	2.54	8.81	50.1	66.3	6.89	6.04	14.9	691	7.39	68.7	3.12	1.37	0.30	0.03	0.01	14.1	9.83	0.40	122	10.3	19.8	2.27	8.53	1.63	0.33	1.74	0.24	1.28	0.25	0.79	0.11	0.75	0.11	1.82	0.28	0.15	0.00	0.00	0.13	3.26	1.24
VS053	83.1	190	8.89	6.88	3.94	17.1	19405	94.6	4.13	29.8	7.67	253	5.76	76.0	1.84	0.26	3.99	0.17	0.35	462	188	0.22	613	8.92	17.2	2.10	8.37	1.73	0.34	1.64	0.23	1.06	0.21	0.60	0.08	0.55	0.08	1.99	0.15	0.41	0.00	0.13	8.18	3.44	2.29
VS058	31.5	160	9.86	9.57	2.61	17.1	278	62.7	5.70	11.9	23.6	279	5.94	56.8	2.48	0.60	0.85	0.11	0.19	6.29	14.8	0.54	257	8.49	16.6	1.91	7.45	1.51	0.32	1.45	0.20	1.05	0.22	0.61	0.09	0.61	0.09	1.53	0.20	0.06	0.00	0.00	0.26	3.08	1.17
VS061	58.4	219	10.6	12.4	3.36	18.1	762	70.6	6.27	48.3	24.1	304	6.80	69.2	2.80	1.19	1.38	0.06	0.10	124	62.3	0.56	229	9.91	19.3	2.25	8.84	1.72	0.33	1.64	0.23	1.18	0.24	0.72	0.10	0.66	0.10	1.80	0.24	0.15	0.00	0.00	0.42	3.70	1.15
VS063	38.9	175	7.95	9.52	4.31	64.3	20513	183	5.43	662	26.4	289	5.68	82.2	2.55	0.49	26.9	0.24	1.64	177	484	0.58	168	8.89	17.7	2.01	7.89	1.49	0.28	1.44	0.20	1.00	0.20	0.59	0.08	0.59	0.09	2.12	0.21	0.13	0.00	0.12	3.25	3.50	0.99
VS073	101	253	13.7	15.5	4.02	10.9	27.9	121	5.37	8.77	8.86	459	8.48	52.7	3.09	0.27	0.09	0.10	0.01	2.32	57.6	0.36	1865	9.93	18.0	2.05	8.26	1.68	0.46	1.52	0.24	1.40	0.28	0.78	0.11	0.79	0.11	1.44	0.20				0.03	2.58	0.83
VS079	26.8	190	8.03	10.1	2.25	20.3	66.3	94.1	4.91	0.53	17.3	291	7.31	110	2.65	1.16	0.14	0.02	0.01	4.36		0.35	115	10.3	19.2	2.17	8.35	1.62	0.23	1.37	0.22	1.20	0.25	0.73	0.11	0.75	0.12	2.92	0.26				0.07	4.51	1.23
VS080	57.3	176	7.12	9.66	2.07	12.1	183	66.3	3.82	1.88	11.2	242	4.85	78.0	1.82	0.78	1.89	0.04	0.05	5.59	28.0	0.23	240	8.18	16.0	1.83	7.04	1.33	0.21	1.18	0.18	0.88	0.17	0.49	0.07	0.45	0.07	2.06	0.14				0.31	3.44	1.44
VS081	43.7	124	12.4	13.1	3.34	13.6	680	78.0	4.69	11.8	21.3	412	6.45	62.4	2.39	1.20	2.07	0.05	0.03	112	37.0	0.59	329	9.55	18.2	2.09	8.26	1.59	0.33	1.42	0.22	1.12	0.22	0.63	0.09	0.60	0.09	1.61	0.18	0.61	0.00	0.01	0.35	3.01	1.12
VS094	31.6	157	10.1	8.12	2.54	16.9	248	62.1	5.66	10.9	23.4	277	5.93	56.8	2.55	0.53	0.77	0.11	0.16	5.78	9.12	0.54	255	9.02	17.4	2.01	7.71	1.48	0.30	1.28	0.20	1.02	0.20	0.58	0.09	0.58	0.09	1.52	0.21	0.05	0.00		0.23	3.31	1.22
VS102	65.9	195	10.0	13.5	2.23	18.8	74.9	59.5	5.02	2.61	7.13	320	5.33	91.2	3.06	0.56	0.94	0.03	0.01	1.91	1.93	0.14	79.8	7.47	14.1	1.58	6.11	1.22	0.25	1.04	0.16	0.86	0.20	0.57	0.08	0.61	0.09	2.31	0.22	0.04	0.00	0.00	0.19	3.06	1.20
VS110	50.1	173	9.98		1.80	16.9	56.5	55.4	4.62		6.19	241	4.36	81.1	2.60	0.93	0.30	0.03	0.01	2.12	0.39	0.11	218	6.10	11.4	1.31	5.10	0.98	0.22	1.02	0.15	0.74	0.16	0.47	0.07	0.53	0.08	2.03	0.18	0.05	0.00		0.03	2.42	1.07
VS112	57.0	189	7.72		2.51	18.3	2128	75.8	4.20	4.07	21.5	304	4.81	49.5	2.01	0.75	2.14	0.16	0.00	49.7	2.84	0.48	157	7.14	13.9	1.65	6.29	1.22	0.25	1.27	0.17	0.84	0.17	0.51	0.07	0.51	0.07	1.32	0.17	0.06	0.00	0.05	0.49	2.66	0.85
VS113	44.0	206	10.3		2.80	12.6	19.1	53.3	5.06	0.21	11.8	758	6.94	129	3.36	0.48	0.54	0.02	0.00	21.9	2.91	0.18	148	13.7	28.1	3.00	12.0	1.89	0.38	2.07	0.26	1.26	0.26	0.78	0.11	0.74	0.11	3.13	0.23	0.05	0.00	0.00	0.07	2.55	0.71
VS115	6.91	97.7	18.4		14.1	12.8	292	80.7	3.95	2.45	13.7	514	6.60	64.0	2.23	2.23	1.09	0.06	0.00	57.8	87.1	0.14	247	6.81	12.0	1.54	6.31	1.29	0.37	1.35	0.21	1.09	0.22	0.65	0.09	0.62	0.09	1.60	0.14	0.26	0.00	0.02	0.18	1.04	0.67
VS116	38.7	151	8.93		2.70	37.1	31354	119	4.85	72.8	23.0	390	6.53	95.6	2.49	0.30	18.8	0.07	0.25	60.7	74.2	0.48	160	10.7	21.4	2.52	10.1	1.94	0.37	1.95	0.26	1.18	0.24	0.71	0.09	0.64	0.10	2.49	0.20	0.10	0.00	0.08	9.70	4.26	1.07
VS119	50.6	172	10.7		3.18	17.7	389	116	5.44	46.8	24.3	370	6.36	74.3	2.56	2.24	2.20	0.13	0.00	256	105	0.55	239	10.2	19.3	2.21	8.63	1.71	0.34	1.45	0.23	1.11	0.22	0.63	0.09	0.63	0.09	1.93	0.21	0.19	0.00	0.01	0.95	3.33	1.51
VS133	78.2	182	9.14		3.92	17.1	19309	101	4.16	31.0	7.52	254	5.87	79.1	1.93	0.24	3.94	0.16	0.34	481	193	0.21	599	9.73	18.7	2.18	8.70	1.74	0.34	1.46	0.23	1.11	0.21	0.58	0.08	0.55	0.08	1.97	0.15	0.44	0.00	0.15	8.25	3.75	2.45
VS138	88.0	178	7.94		2.91	17.7	14229	114	4.28	23.1	10.6	309	5.74	74.6	2.05	0.41	8.51	0.07	0.14	55.9	107	0.23	448	10.2	20.1	2.33	9.14	1.78	0.34	1.44	0.22	1.06	0.21	0.59	0.08	0.55	0.08	1.96	0.17	0.32	0.00	0.27	2.48	3.66	2.55
VS141	7.28	87.9	22.9		10.9	12.3	186	77.2	4.77	3.82	12.9	655	8.21	78.7	3.15	2.68	0.74	0.04	0.01	40.6	35.5	0.17	246	9.13	16.7	1.98	8.25	1.56	0.43	1.40	0.26	1.34	0.28	0.81	0.11	0.74	0.11	1.88	0.18	0.31	0.00	0.03	0.11	1.55	0.80
Soda ash lead glass																																														
VS032	9.21	49.8	7.67	3.45	3.50	7.81	338	54.2	1.77	109	3.21	51.8	2.07	14.7	0.81	0.70	27.9	0.23	0.00	1591	482	0.17	78.3	2.55	4.70	0.51	2.01	0.39	0.10	0.38	0.07	0.35	0.07	0.22	0.03	0.23	0.03	0.39	0.07	0.49		0.01	23.2	0.74	0.25
VS054	20.8	58.7	11.8	8.56	10.5	17.0	1829	177	2.65	58.8	5.38	89.6	3.02	19.2	1.07	1.01	33.9	0.33	0.00	951	381	0.28	76.0	3.48	6.51	0.77	3.21	0.72	0.17	0.73	0.11	0.55	0.11	0.30	0.04	0.31	0.04	0.51	0.07	1.00	0.00	0.02	20.1	0.98	0.38
VS108	15.9	60.4	9.55		2.73	44.7	14011	69.2	2.38	50.2	4.52	72.9	2.77	20.8	1.11	0.28	49.1	0.16	0.00	552	361	0.20	74.6	3.45	6.63	0.76	3.06	0.65	0.17	0.70	0.10	0.52	0.11	0.31	0.04	0.29	0.04	0.55	0.08	0.17	0.00	0.88	34.2	1.05	0.27
VS123	10.8	57.0	14.0		5.30	23.8	617	55.2	2.78	181	4.07	78.7	3.01	17.6	1.21	0.73	47.9	0.12	0.00	2291	757	0.27	47.2	3.97	7.30	0.81	3.27	0.72	0.16	0.60	0.11	0.54	0.12	0.32	0.05	0.31	0.05	0.49	0.09	0.78	0.00	0.03	20.8	1.11	0.44
VS124	13.7	47.0	13.2		6.63	14.8	951	56.1	2.89	118	6.43	62.6	3.22	22.5	1.26	0.67	32.0	0.33	0.00	2966	418	0.43	68.9	4.32	7.88	0.89	3.53	0.78	0.17	0.67	0.12	0.62	0.12	0.34	0.04	0.31	0.05	0.60	0.09	0.80	0.00	0.04	17.8	1.24	0.43
VS139	8.90	35.5	4.54		0.87	10.4	5766	42.0	0.94	42.3	2.42	59.7	1.48	11.2	0.54	0.35	52.0	0.12	0.00	894	824	0.11	22.7	1.95	3.66	0.42	1.61	0.40	0.07	0.29	0.05	0.28	0.05	0.15	0.02	0.16	0.02	0.31	0.04	0.05	0.00	0.03	30.1	0.59	0.14
Outliers																																														
VS012	134	126	10.1	9.57	2.48	12.0	20040	56.7	4.97	15.7	14.5	267	4.80	32.1	2.26	0.31	15.3	0.06	0.02	23.8	29.1	0.36	98.1	6.94	13.7	1.55	6.17	1.21	0.24	1.36	0.19	0.87	0.18	0.54	0.07	0.47	0.07	0.87	0.17	0.06	0.00	0.22	2.62	2.44	2.07
VS021	29.6	236	6.20	11.7	4.47	6.56	24.2	105	4.44	17.6	4.10	367	7.70	25.3	1.81	0.28		0.05	0.01	0.35	82.7	0.40	2535	6.08	11.3	1.31	5.19	1.19	0.60	1.17	0.20	1.13	0.23	0.65	0.08	0.54	0.07	0.68	0.12	258			0.02	1.58	0.46
VS027	48.6	174	6.49	9.03	4.85	10.6	28.9	86.8	3.63	10.1	9.16	482	14.9	16.8	0.97	0.43	0.05	0.15	0.02	0.40	74.4	0.34	1693	6.10	11.4	1.30	5.18	1.26	0.47	1.24	0.28	1.86	0.46	1.45	0.21	1.62	0.23	0.42	0.06	133	0.00	0.00	0.03	1.37	0.37
VS091	29.5	183	9.90	11.5	2.41	10.2	14.3	81.7	3.81	1.51	5.01	836	5.27	47.7	2.12	0.54	0.07	0.05	0.01	0.80	0.65	0.12	116	6.85	13.2	1.49	5.75	1.15	0.24	1.03	0.17	0.88	0.20	0.55	0.08	0.51	0.08	1.25	0.16	0.03		0.00	0.02	2.11	1.14
VS092	124	162	23.9	25.1	5.98	17.1	154	108	11.3	9.55	30.4	446	11.7	79.3	5.26	0.39	1.32	0.14	0.02	64.8	23.4	0.94	209	17.7	35.4	3.92	15.1	3.00	0.57	2.63	0.41	2.14	0.42	1.18	0.17	1.13	0.17	2.13	0.39	0.29	0.00	0.02	0.86	6.55	3.63
