# Supplementary material for: Glass import and production in Hispania during the early medieval period: The glass from Ciudad de Vascos (Toledo)
Source: PLoS One. 2017 Jul 26;12(7):e0182129. doi: 10.1371/journal.pone.0182129 (PMC5529010; doi:10.1371/journal.pone.0182129)
Supplement: S2 Table — (PDF) [file pone.0182129.s002.pdf]

S2 Table. LA-ICP-MS data of glass standards in comparison with published values

Major and minor elements [wt %]

	Li_2O	B_2O_3	Na_2O	MgO	Al_2O_3	SiO_2	P_2O_5	Cl	K_2O	CaO	TiO_2	V_2O_5	MnO	Fe_2O_3	CoO	NiO	CuO	Rb_2O	SrO	ZrO_2	SnO_2	Sb_2O_3	BaO	PbO	Bi
Corning A (n=12)	0.01	0.20	13.79	2.57	0.94	67.00	0.11	0.13	2.84	5.66	0.75	0.01	1.02	1.12	0.17	0.02	1.17	0.01	0.10	0.01	0.17	1.62	0.46	0.06	0.001
relative σ [%]	2.40	2.74	1.61	2.00	2.85	0.50	5.26	2.18	2.23	0.72	2.10	1.69	1.12	1.28	0.98	0.60	0.75	0.82	0.74	3.44	1.13	2.65	1.09	1.32	0.740
Vicenzi [1]	0.01	0.20	14.30	2.66	1.00	66.56	0.13	0.10	2.87	5.03	0.79	0.01	1.00	1.09	0.17	0.02	1.17	0.01	0.10	0.01	0.19	1.58	0.56	0.073[4]	0.00
accuracy [%]	-6.78	-1.00	3.55	3.56	6.17	-0.66	11.86	-25.46	1.01	-12.47	5.08	-8.74	-2.45	-2.62	0.64	-13.45	0.41	4.10	-3.99	-7.82	12.22	-2.76	18.16	16.21	13.550
NIST 612 (n=12)			13.45		2.13	72.48				11.63															
relative σ [%]			1.37		2.86	0.44				0.84															
Jochum [2]			14.00		2.06	71.70				11.93															
Hollocher [3]			13.16		2.32	72.17				12.09															
accuracy [%]			3.90 / -2.23		3.19 / 8.37	-1.09 / -0.43				2.54 / 3.83															

Trace elements [ppm]

	V	Cr	Mn	Co	Ni	Cu	Zn	Ga	As	Rb	Sr	Y	Zr	Nb	Mo	In	Sn	Sb	Cs
NIST 612 (n=12)	38.35	37.59	40.63	35.06	37.82	36.42	36.85	36.73	33.16	32.12	76.88	38.15	38.00	35.40	33.06	36.99	34.03	33.93	41.26
relative σ [%]	1.68	5.72	0.92	0.59	1.52	0.84	2.21	1.04	3.63	0.55	0.67	2.35	3.05	2.95	1.88	0.26	0.61	3.60	0.80
Jochum / Hochlocher	39.90	36.26	39.40	34.82	37.90	38.70	41.20	34[3]	36.80	31.07	78.51	40.14	40.36	41.54	35.79	41[3]	40.90	36.40	42.14
accuracy [%]	3.89	-3.67	-3.12	-0.70	0.20	5.89	10.55	-8.04	9.89	-3.37	2.08	4.97	5.85	14.78	7.63	9.78	16.79	6.77	2.08

	Ba	La	Ce	Pr	Nd	Sm	Eu	Gd	Tb	Dy	Ho	Er	Tm	Yb	Lu	Hf	Ta	Pb	Bi	U
NIST 612 (n=12)	36.40	38.76	39.34	37.55	35.26	37.08	36.59	34.01	37.89	34.47	38.57	36.00	36.19	38.61	37.16	35.57	31.76	32.49	29.96	38.38
relative σ [%]	1.18	6.99	6.78	4.77	3.16	2.00	1.18	7.28	1.76	1.72	1.97	1.77	1.66	1.33	1.85	3.19	2.44	2.68	0.85	4.22
Jochum / Hochlocher	39.37	34.65	37.25	37.31	34.96	37.15	34[3]	38.56	39.96	36.15	38.69	38.86	36[3]	39.84	35[3]	37.13	38.17	38.60	40[3]	37.68
accuracy [%]	7.54	-11.86	-5.62	-0.64	-0.87	0.19	-7.62	11.79	5.17	4.64	0.31	7.37	-0.52	3.10	-6.17	4.20	16.79	15.82	25.10	-1.85

1. Vicenzi EP, Eggins S, Logan A, Wysoczanski R. Microbeam characterization of corning archeological reference glasses: new additions to the smithsonian microbeam standard collection. Journal of Research of the National Institute of Standards and technology. 2002;107(6):719.
2. Jochum KP, Weis U, Stoll B, Kuzmin D, Yang Q, Raczek I, et al. Determination of reference values for NIST SRM 610–617 glasses following ISO guidelines. Geostandards and Geoanalytical Research. 2011;35(4):397-429.
3. Hollocher K, Ruiz J. Major and trace element determinations on NIST glass standard reference materials 611, 612, 614 and 1834 by inductively coupled plasma-mass sectrometry. Geostandards Newsletter. 1995;19(1):27-34.
4. Wagner B, Nowak A, Bulska E, Hametner K, Günther D. Critical assessment of the elemental composition of Corning archeological reference glasses by LA-ICP-MS. Analytical and bioanalytical chemistry. 2012;402(4):1667-1677.
